# CCR1 and CCR5 mediate cancer-induced myelopoiesis and differentiation of myeloid cells in the tumor

**DOI:** 10.1136/jitc-2021-003131

**Published:** 2022-01-21

**Authors:** Serena Zilio, Silvio Bicciato, Donald Weed, Paolo Serafini

**Affiliations:** 1 Department of Microbiology and Immunology, Sylvester Comprehensive Cancer Center, University of Miami, Miller School of Medicine, Miami, Florida, USA; 2 Department of Life Sciences, University of Modena and Reggio Emilia, Modena, Italy; 3 Department of Otolaryngology, Sylvester Comprehensive Cancer Center, University of Miami, Miller School of Medicine, Miami, Florida, USA

**Keywords:** immunomodulation, myeloid-derived suppressor cells, neutrophil infiltration, receptors, antigen, immunity, innate

## Abstract

**Background:**

Cancer-induced ‘emergency’ myelopoiesis plays a key role in tumor progression by inducing the accumulation of myeloid cells with a suppressive phenotype peripherally and in the tumor. Chemokine receptors (CCRs) and, in particular, CCR1, CCR2, CCR5, and CCR7 are emerging as key regulators of myeloid cell trafficking and function but their precise role has not been completely clarified yet because of the signal redundancy, integration, and promiscuity of chemokines and of the expression of these CCRs on other leukocyte subsets.

**Methods:**

We used the 4PD nanoparticle for the in vivo targeted silencing of CCR1, CCR2, CCR5, and/or CCR7 in the myeloid cells of tumor bearing mice to evaluate the effect of treatments on tumor growth, myeloid cell trafficking and polarization. We used flow and image cytometry and functional assays to monitor changes in the tumor microenvironment and depletion experiments and immune deficient mice to determine the role of Ly6G^+^cells during tumor progression. We further evaluated in vitro the impact of chemokine receptor inhibition and tumor derived factors on myeloid cell differentiation from mouse and human hematopoietic stem and precursors cells (HSPCs) using flow cytometry, transcriptome analysis, cytokines beads arrays, functional assays, and mice deficient for CCR1 or CCR5.

**Results:**

4PD-mediated in vivo silencing of CCR1 and CCR5 on myeloid cells and myeloid precursors was necessary and sufficient to inhibit tumor progression. Functional studies indicated that this antitumor effect was not mediated by alteration of myeloid cell chemotaxes but rather by the repolarization of polymorphonuclear myeloid-derived suppressor cells (MDSCs) into tumoricidal neutrophils. Transcriptome functional and cytokine analysis indicated that tumor derived factors induced CCL3 and CCL4 in HSPCs that, through the autocrine engagement of CCR1 and CCR5, induced HSPCs differentiation in MDSCs. These finding were confirmed across mice with different genetic backgrounds and using HSPCs from umbilical cord blood and peripheral blood of patients with cancer.

**Conclusions:**

Our data support the notion that CCR1 and CCR5 and their ligands are a master immunological hub activated by several tumor derived factors. Activation of this pathway is necessary for the differentiation of MDSCs and protumoral macrophages.

## Background

Myeloid cells in the tumor micro-environment and macro-environment can either promote or restrain tumor progression depending on their intrinsic polarization. Type-2 myeloid cells such myeloid-derived suppressor cells (MDSCs), inflammatory monocytes, and ‘M2’ macrophages promote immune exclusion and evasion, resistance to therapy, angiogenesis, and neoplastic cell proliferation and spreading, and are independent prognostic factors for overall survival in many human malignancies.^1–3^ Conversely, type-1 myeloid cells can exert a direct tumoricidal activity, mediate the action of antibody therapies, and stimulate the adaptive antitumor immunity.^4–6^ These cells are associated with better survival in some cancer types.^7 8^ Thus, modulation of myeloid cell composition can have important repercussions on cancer progression and response to treatment.

Inflammatory chemokines such as CCL2, 3, 4, 5, 7, and 21, present in most human malignancies, modulate both neoplastic cell biology and the composition of tumor-infiltrating myeloid cells.^9^ While the CCL2 receptor chemokine receptor (CCR)2 is the dominant receptor for recruiting myelomonocytic cells,^10^ the CCL3 and CCL4 receptors CCR1 and CCR5 are also associated with MDSC accumulation,^11–14^ and the CCL21 receptor CCR7 can promote the formation of intratumoral tolerogenic lymph node-like structures.^15^ The dissection of the individual roles of CCR1, 2, 5, and 7, however, is hindered by the coexpression of these receptors in the same myeloid subsets,^16^ by their capacity to bind to the same chemokine and to trigger similar intracellular signals,^17^ and by the possible integration of downstream signaling pathways.^18^ Each chemokine can bind multiple receptors and a receptor can be activated by different chemokines, allowing for signal redundancy, robustness, integration, and synergy. This makes the understanding of their individual contribution to myeloid cells in tumor host function difficult.^19^ Additionally, CCR 1, 2, 3, and 5 occupy a discrete and tight (165 kb) chromosomal locus (Chr9: 123962126–124127183 bp) and are expressed by most leukocyte subsets, making the in vivo study of their signal integration or redundancy difficult using the available mouse knock-out approaches.

To overcome these difficulties, we used the 4PD nanoplatform that targets tumor-infiltrating myeloid cells to determine the relative contributions of CCR1, 2, 5, and 7 silencing on myeloid cell function in tumor hosts.^20^ Our study provides evidences for a synergistic and redundant role of CCR1 and 5 in mediating cancer-induced myelopoiesis and myeloid cell protumoral polarization. We show that tumor derived factors prime hematopoietic stem and precursors cells (HSPCs) to secrete CCL3 and 4 that promote HSPC differentiation into protumoral MDSCs via CCR1 and CCR5 signaling.

## Methods

All material and reagents are summarized in online supplemental table 1. Flow and image cytometry, qRT-PCR, CBA, cell isolation, and functional assays are described in online supplemental methods.

10.1136/jitc-2021-003131.supp1 Supplementary data

### Cell lines

4T1, CT26, TSA, MCA203, DA3, A20, B16Lu8, HEK293, MDA-MB-231, cell lines were obtained from the American Type Culture Collection(ATCC), used within six passages from the original shipment, and maintained in complete media (online supplemental table 1) with 2-β-mercaptoethanol (20 µM). MDA-BoM-1833, and MDA231-LM2-4175 (a kind gift from Dr Lippman, University of Miami), 4T1HAThy1.1luciferase (a kind gift from Dr Borrello Johns, Hopkins University) were used within 10 passages from acquisition and maintained in complete media. B4B8 (a kind gift from Dr Thomas, University of Miami) were maintained in keratinocyte-SFM media supplemented with L-glutamine (2 mM), BPE (50 µg/mL) and EGF (5 pg/mL). Cells were annually tested via PCR for the presence of common pathogens and authenticated at the end of the project by STR analysis. Tumor conditioned media (TCM) was generated by admixing three parts of 0.2 µm filtered supernatant from the indicated cells (plated (3×10^6^) 4 days early in T75 flask in 20 mL of complete media) with seven parts of complete media.

### Human primary cells

Blood from patients (median age 59 years, range 49–72) with recurrent stage 3 or 4 head and neck squamous cell carcinoma (HNSCC) of the oral cavity or oropharynx undergoing salvage surgery were collected at the time of surgery. Umbilical cord blood (UCB) units from female and male newborn babies (2 days old) underwent Ficoll separation and red blood cell lysis.

### Antagonists, shRNAs, and 4PD

B×471 and maraviroc (Sigma-Aldrich, 10 µM and 5 µM, respectively) were added to the culture on day 0 and 3. 4PD^20^ (Kerafast) was complexed with short hairpin RNAs (shRNAs) (online supplemental table 1) at a 10:1 N/P-ratio following manufacturer instructions.

### Mice and tumor experiments

8–10 weeks old BALB/c, C57BL/6J, and NSG mice were purchased from JAX laboratory, allowed to acclimate for at least 1 week, and maintained in the pathogen-free animal facilities at the University of Miami. Ear-tagged mice were randomized after tumor inoculation. BALB/c mice were injected orthotopically with the 3×10^5^ 4T1 cells, or subcutaneously (s.c.) with 5×10^5^ CT26, 2×10^6^ B4B8, or 5×10^5^ TSA cells. C57BL/6 mice were injected s.c. with 5×10^5^ MCA203 cells. Mice were injected intravenously three times a week with shRNAs (0.5 ug/g of each) loaded 4PD and euthanized when tumor reached ~1.2 cm of diameter or if signs of treatment or tumor related toxicity were evident as per IACUC guidelines. Tumors are expressed as volume (V)=L×S^2^×0.52 where L is the largest diameter, and S is the perpendicular one.

CCR5^-/-^C57BL/6, Cl4-Tg(TcraCl4, TcrbCl4)1Shrm/ShrmJ, and OT1-Tg(TcraTcrb)1100Mjb/J mice and CCR1^−/−^C57BL/6 mice (a kind gift from Dr P Murphy, NIH) were bred in our facility. We used Cl4 and OT1 mice recognizing the K^d^-restricted HA_518–526_ peptide and the K^b^ restricted OVA_257–264_ peptide, respectively, for suppressive assays. Depletion experiments were performed by intrapertioneally injection of the 1A8 rat-anti-mouse Ly6G antibody (10 µg/g) or IgG2a isotype control.

### Tumor specimens

Mouse and human tumor specimens were processed within 1 hour from resection, cut in small pieces (~2×2 mm^2^), washed two times with phosphate-buffered saline (PBS), incubated for 10–20’ at 37°C with 5 volumes of PBS containing collagenase IV (10 mg/mL), MgCl_2_ (100 µM), and CaCl_2_ (100 µM), and minced by passing the mixture through a 3 mL needleless syringe every 10’. Reaction was stopped with 3 volumes of PBS-EDTA (2 mM), and cells were filtered with a cell-strainer and washed with PBS.

### Statistics

All values depicted represent mean±SD of biological replica. Statistical tests (one-way, two-way, or three-way, analysis of variance (ANOVA) followed by Holm-Sidak or Bonferroni test for multiple pairwise comparison or Student’s t-test) were applied in a two-sided, unpaired fashion after normality was evaluated by the Shapiro-Wilk test. ANOVA on ranks followed by Tukey test for multiple comparison was used for those data that failed the normality test. The variance was similar between experimental groups in each experiment unless otherwise stated. In vitro analyses and in vivo experiments were repeated two to five times to ensure reproducibility and cumulative data are shown. Log-rank test was used for survival analysis followed by pairwise multiple comparison procedures (Holm-Sidak method). No experimental data point was excluded from analysis. Sample size was determined by power analysis using effect size determined by pilot experiments or prior experience of the authors. Statistical analyses were performed with Sigmaplot.

### Study approval

All animal experiments were conducted under a protocol approved by the Division of Veterinary Resources and the Institutional Animal Care & Use Committee of the University of Miami.

## Results

### The 4PD nanoplatform allows to simultaneously silence multiple chemokine receptors *in vivo*

**Figure 1:**
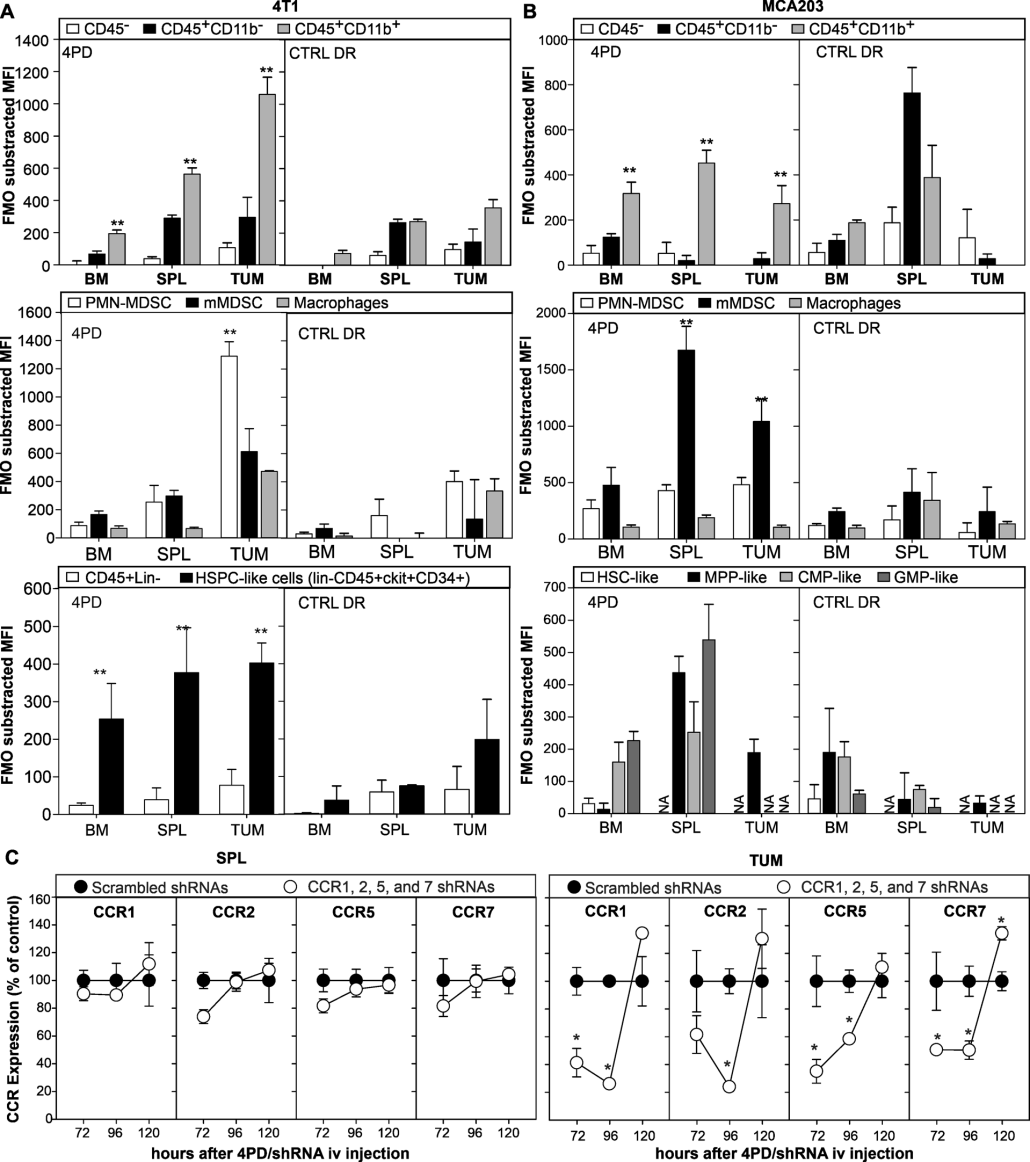
4PD allows the simultaneous targeted silencing of multiple chemokine receptors. BALB/c (A) or C57BL/6 (B) mice were challenged with the 4T1 mammary carcinoma orthotopically or the MCA203 fibrosarcoma subcutaneously, respectively. When tumor reached approximately 0.5 cm in diameter, mice were injected intravenously with 4PD loaded with AF555 siRNA. Two hours later, 4PD binding to the indicated myeloid subsets was evaluated in the bone marrow, spleen, and tumor by flow cytometry. **P<0.001 in Bonferroni multiple comparison test across leukocyte subsets in each tissue post three-way ANOVA using tissue, leukocyte subset and treatment as factors. (C) 4T1 bearing BALB/c mice were injected intravenously with 4PD loaded with an equimolar mixture of shRNAs specific for CCR1, CCR2, CCR5, and CCR7. Expression of these chemokine receptors was evaluated by qRT-PCR at the indicated time on CD11b^+^cells isolated from tumors and spleens. **P<0.001 in two-way ANOVA vs control. ANOVA, analysis of variance; CCR, chemokine receptors; iv, intravenous; mMDSC, monocytic MDSC; MDSC, polymorphonuclear myeloid-derived suppressor cell; shRNA, short hairpin RNA; siRNA, small inhibitory RNA.

qRT-PCR analysis of CD11b^+^cells from the tumor or the spleen of 4T1 bearing mice indicates that CCR1, 5 and 7 are significantly upregulated at the tumor whereas CCR2 is highly expressed on myeloid cells from both tissues (online supplemental figure 1). To understand the role of these receptors on myeloid cell composition and function at the tumor site, we employed the previously characterized 4PD nanoplatform to silence in vivo CCR1, 2, 5 and 7.^20^ On injection in BALB/c mice bearing the 4T1 carcinoma, this nanoparticle recognized preferentially (p<0.001) tumor-infiltrating myeloid cells as determined by three way-ANOVA using tissues, leukocyte subsets (CD45^−^ vs CD45^+^CD11b^−^ vs CD45^+^CD11b^+^), and treatment as factors. Further analysis revealed that 4PD accumulated mostly on tumor-infiltrating polymorphonuclear (PMN)-MDSC (p<0.001). In sharp contrast, the control dendrimer conjugated with random peptides were less specific (figure 1A). Interestingly, we observed also 4PD binding to lineage negative CD34^+^ckit^+^ hematopoietic precursors in the bone marrow, spleen and tumor suggesting that this platform might be used to modulate gene expression on HSPCs. We repeated the experiment in the C57BL/6 MCA203 fibrosarcoma model characterized by fewer MDSCs in the spleen and in the tumor. In this setting as well, 4PD showed a preferential binding to myeloid cells (p=0.003) in all tissues evaluated. As we previously showed in the B16 model, in the MCA203 model, 4PD bound preferentially monocytic MDSCs (mMDSCs) (p<0.001) in both spleen and tumor. This can be explained by the heterogeneity of myeloid cell subsets across tumor models and mice strains.^20^ Also in the MCA203 model, 4PD recognized HSPCs-like cells and, in particular, cells resembling multipotent progenitors (MPPs) and granulocyte-monocyte progenitors (GMPs) in the spleen and MPP-like cells in the tumor (figure 1B).

We then evaluated the ability of the 4PD nanoplatform to silence multiple CCRs in myeloid cells in time course experiments. To this aim, we injected 4PD loaded with an equimolar mixture of shRNAs against CCR1, 2, 5, and 7 intravenously to 4T1 tumor bearing mice. This treatment silenced these genes for 3–4 days in tumor-infiltrating myeloid cells but not in the splenic counterpart (figure 1C) nor in CD11b negative cells (not shown).

### Simultaneous silencing of CCR1 and CCR5 on myeloid cells in vivo inhibits tumor progression

**Figure 2:**
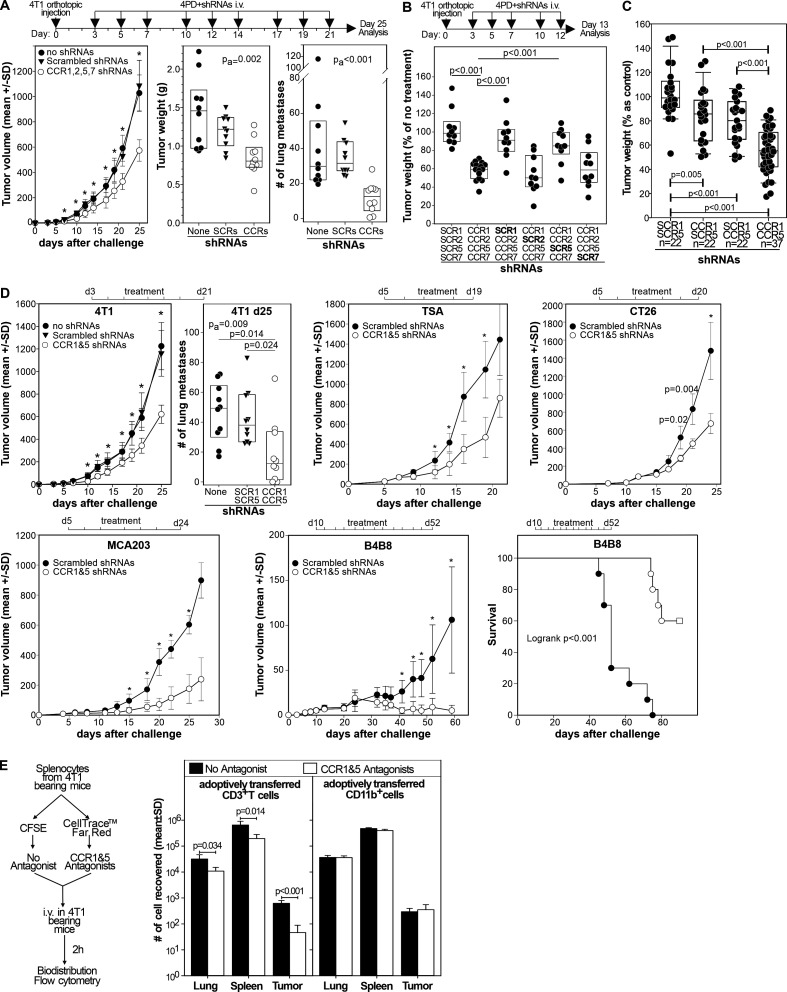
Targeted CCR1 and CCR5 silencing limits tumor progression by chemotaxis independent mechanisms. (A) BALB/c mice (n=10) were injected orthotopically with the 4T1 mammary tumor and, starting 3 days after challenge, treated three times a week with 4PD loaded with the indicated shRNAs. Twenty-five days after challenge mice were euthanized primary tumors weighted and lung metastases counted. Data are cumulative of two independent experiments (B) mice (n=10–16) treated as in (A) were euthanized 13 days after challenge and tumor weighted. Data are cumulative of three independent experiments and expressed as percentage of control (untreated 4T1 bearing mice). (C) Mice (n=22–37), challenged 3 days before with 4T1 cells, were intravenously treated three times a week with 4PD loaded with (i) scrambled shRNAs, (ii) CCR1 and scrambled_CCR5_ shRNAs, (iii) CCR5 and scrambled_CCR1_ shRNAs, or (iv) CCR1 and CCR5 shRNAs. Tumor weight was measured 13 days after challenge. Data are cumulative of five independent experiments. (D) Mice (n=10) bearing the indicated tumors were treated intravenously three times a week with 4PD loaded with the indicated shRNAs. Tumor progression was followed. Significant differences were evaluated by two-ways ANOVA using ‘time’ and ‘treatment’ as factors. *=P<0.001 within each time point in a post-hoc Holm-Sidak test for multiple pairwise comparison. Data derived from two independent experiments in each model. (E) Splenocytes from 4T1 tumor bearing mice were divided in two aliquots and either labeled with CellTrace Far Red and pulsed with maraviroc and B×471 or labeled with CFSE and left untreated. 10^7^ cells from a 1:1 mixture of the two aliquots were injected intravenously in 4T1 bearing recipient mice. Transferred CellTrace Far Red^+^ (antagonists) or CSFE^+^ (no antagonists) cells were enumerated among the CD3^+^ or CD11b^+^ leukocytes by flow cytometry 2 hours after injection. ANOVA, analysis of variance; CCR, chemokine receptors; CFSE, Carboxy Fluorescein Succinimidyl Ester; shRNA, short hairpin RNA.

Once the capacity of 4PD to bind and transfect myeloid cells in vivo with multiple shRNAs was determined, we evaluated the effect of chronic CCR1, CCR2, CCR5, and CCR7 silencing on tumor progression. Systemic administration of 4PD loaded with shRNAs specific for CCR1, 2, 5 and 7 to 4T1 bearing mice significantly (p<0.001) delayed tumor progression and decreased the number of lung metastases, whereas the corresponding scrambled shRNAs (SCR1, 2, 5, and 7) had no effect (figure 2A). To evaluate which CCRs were responsible for the antitumor effect, 4T1 bearing mice were given 4PD loaded with shRNAs against: (i) CCR1, 2, 5, and 7, (ii) scrambled shRNA1, CCR2, 5, and 7, (iii) CCR1, scrambled shRNA2, CCR5 and 7, (iv) CCR1 and 2, scrambled shRNA5, and CCR7, (v) CCR1, 2, and 5 and scrambled shRNA7 or (vi) scrambled shRNAs1, 2, 5 and 7. Again, simultaneous silencing of all four CCRs resulted in an important antitumor effect that was lost when either CCR1 or CCR5 were omitted from the shRNA mixture. In contrast, the absence of CCR2 or CCR7 shRNAs did not impair the therapeutic effect (figure 2B). Then, we evaluated whether silencing of CCR1 and/or CCR5 was sufficient to inhibit tumor growth (figure 2C). While silencing of either CCR1 or CCR5 gave modest results, silencing both receptors significantly (p<0.001) reduced tumor size. We observed no effect when mice were treated with scrambled shRNAs. To determine whether this effect was reproducible in other models, we challenged mice with the 4T1 tumor, the TSA breast carcinoma, the CT26 colon carcinoma, the B4B8 squamous cell carcinoma (figure 2D), or the MCA203 fibrosarcoma. Once tumors become palpable, mice were treated with shRNAs against CCR1 and 5. In all these models, chronic targeted silencing of CCR1 and CCR5 significantly reduced tumor growth and the lung metastases in the 4T1 model (online supplemental figure 2). Interestingly, in the slow growing B4B8 carcinoma, treatment induced tumor rejection in approximately 60% of the mice.

Finally, we evaluated whether CCR1 and CCR5 blockade inhibited myeloid cell trafficking to the tumor (figure 2E). Briefly, we divided splenocytes from 4T1 bearing mice in two aliquots. One was pulsed with maraviroc and B×471 (specific antagonists of CCR5 and CCR1, respectively) and labeled with CellTrace Far Red dye, the other one was left unpulsed and labeled with Carboxy Fluorescein Succinimidyl Ester(CFSE). The two aliquots were admixed at one to one ratio and injected intravenously in 4T1 bearing mice. Two hours later, we evaluated the biodistribution of donors’ CD3^+^ and CD11b^+^ leukocytes by flow cytometry. While pretreatment with CCR1 and CCR5 antagonists significantly reduced the T cell chemotaxes to the lungs, spleen, and tumor, we did not observe any difference on the biodistribution of CD11b^+^ myeloid cells (figure 2E). This argues against a role of CCR1 and CCR5 on myeloid cell trafficking to the tumor. These data suggest that CCR1 and CCR5 signaling in myeloid cells promotes tumor progression by a mechanism that might be unrelated to their migration.

### CCR1 and CCR5 silencing alters myeloid cell phenotype in the tumor microenvironment

**Figure 3:**
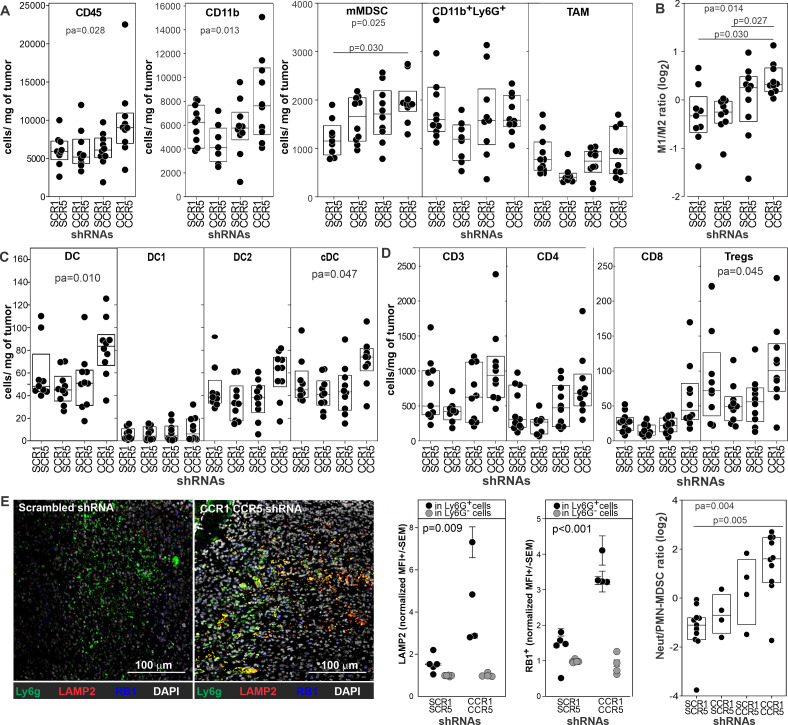
Targeted CCR1 and CCR5 silencing modulates the tumor microenvironment. Tumors from 4T1 bearing mice (n=10) treated with 4PD loaded with (i) scrambled shRNAs, (ii) CCR1 and scrambled_CCR5_ shRNAs, (iii) CCR5 and scrambled_CCR1_ shRNAs, or (iv) CCR1 and CCR5 shRNAs were analyzed by multicolor flow cytometry and data reported as cell density. Gating strategies are depicted in online supplemental figure 2. (A) Myeloid subsets. (B) M1/M2 macrophage ratio. (C) DC subsets. (D) T cell subsets. (E) Tumor sections from the mice (n=5) treated with scrambled or CCR1 and CCR5 shRNAs were stained with DAPI (white) and antibodies against Ly6G (green), Lysosomal Associated Membrane Protein 2 (red) and retinoblastoma 1 (blue), scanned, and analyzed by image cytometry. CCR, chemokine receptors; cDC, conventional dendritic cell; shRNA, short hairpin RNA.

Since CCR1 and CCR5 targeted silencing mediates a significant antitumor effect, we evaluated its action on the tumor microenvironment (figure 3). We treated 4T1 bearing mice with 4PD loaded with shRNA against CCR1 and/or CCR5 or corresponding scrambled shRNAs as control. Thirteen days after challenge, we characterized the tumor microenvironment by flow and image cytometry and tested myeloid cells functionally in suppressive and tumor co-culture assays. Flow cytometry (online supplemental figure 3) showed that silencing of both CCR1 and CCR5 slightly increased the intratumoral density of CD45^+^leukocytes and CD11b^+^myeloid cells whereas did not change the number of macrophages, mMDSCs, and CD11b^+^Ly6G^+^ populations (figure 3A). Analysis of Tumor associated macrophage (TAM) subsets using the major histocompatibility complex (MHC) class II and CD206 markers revealed a significant (p=0.014) change in the polarization of this subset toward a M1-like phenotype (figure 3B) and a modest increase (p=0.010) in the concentration of dendritic cells (DCs) and conventional (c)DCs (p=0.047) in particular (figure 3C). Surprisingly, we did not observe changes in the density of the overall lymphocyte nor in the CD4 and CD8 subsets (figure 3D), arguing against a prominent role of T cells in the observed antitumor effect. We observed no changes when only either of the chemokine receptors was silenced (figure 3).

Since CD11b^+^Ly6G^+^ cells are the largest myeloid subset in this model and they include both ‘classical’ antitumoral neutrophils (NeuT) and protumoral PMN-MDSCs,^21^ that cannot be easily resolved by flow cytometry, we evaluated their phenotype by image cytometry (figure 3E and online supplemental figure 4) using antibodies against Ly6G, Lysosomal Associated Membrane Protein 2 (LAMP2), and retinoblastoma (RB1). While Ly6G identified both PMN-MDSCs and neutrophils, LAMP2 and retinoblastoma allow the discrimination of these two polymorphonuclear subsets.^22^ Ly6G^+^ cells in the tumor of control mice were mostly RB1 and LAMP2 negative (figure 3E) suggesting a PMN-MDSC phenotype.^22^ In sharp contrast, Ly6G^+^ cells in the tumor of mice silenced for CCR1 and CCR5 had a high expression of RB1 and LAMP2 consistent with a ‘classical’ neutrophil phenotype. Taken together, these data suggest that targeted inhibition of CCR1 and CCR5 on myeloid cells alters the tumor microenvironment by reducing the number of PMN-MDSCs and M2 macrophages and by increasing the concentration of cells with a phenotype consistent with classical neutrophils.

### CCR1 and CCR5 targeted silencing changes the function of tumor-infiltrating myeloid cells

**Figure 4:**
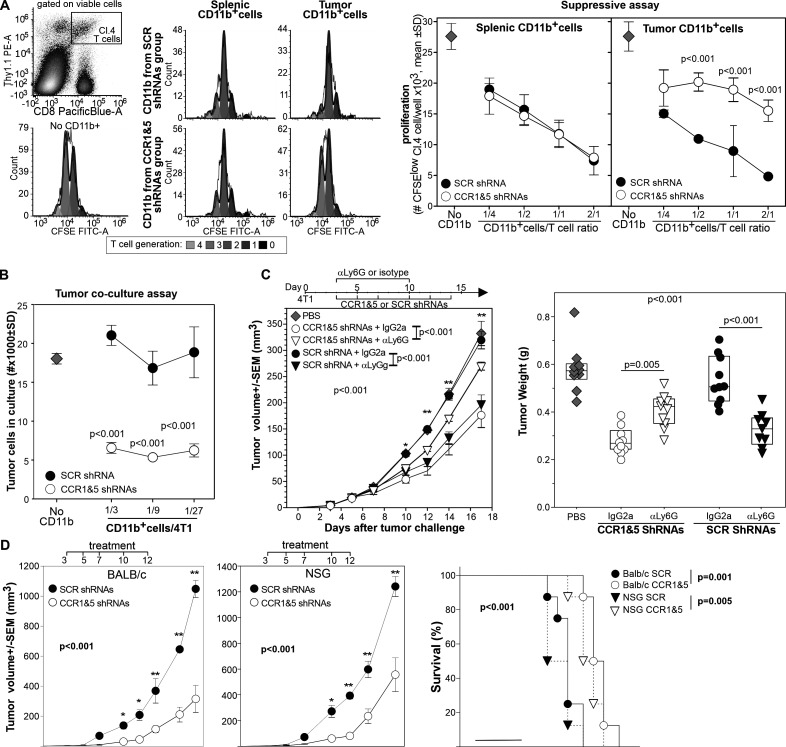
In vivo silencing of CCR1 and CCR5 changes the function of tumor-infiltrating myeloid cells from immunosuppressive to tumoricidal. (A) CD11b^+^cells from the spleen or tumor of 4T1 bearing mice treated with scrambled or CCR1 and CCR5 shRNAs were incubated with HA-specific, CFSE-labeled T cells stimulated with the relevant peptide. Proliferation was evaluated 3 days later by flow cytometry, the number of proliferating clonotypic T cells per well is reported. (B) CD11b^+^cells from the tumor of mice treated with scrambled or CCR1 and CCR5 specific shRNAs were incubated at different ratio with 4T1-luciferase cells. Neoplastic cell number was evaluated 24 hours later by luciferase. *=P<0.001. (C) 4T1 bearing mice (n=10) were treated intravenously with shRNAs against CCR1 and CCR5 or scrambled shRNAs and treated with either rat anti-Ly6G depleting antibody or isotype control as depicted. Tumor progression was monitored, and tumor weight measured 18 days after challenge. (D) BALB/c or NSG mice (n=8/group) were injected orthotopically with 4T1 tumor and treated intravenously with 4PD loaded with scrambled or CCR1 and CCR5 shRNAs as indicated. *: P<0.01; **p<0.001. CCR, chemokine receptors; CFSE, Carboxy Fluorescein Succinimidyl Ester; HA, hemagglutinin; shRNA, short hairpin RNA.

Having observed the changes in the tumor-infiltrating PMNs, we performed functional assays to determine if these changes translated into differences in myeloid cell suppressive or tumoricidal activity. We purified CD11b^+^ myeloid cells from the spleen and the tumor of mice treated with CCR1 and CCR5 shRNAs or with scrambled shRNAs and tested their suppressive activity against HA specific, CD8^+^ T cells stimulated with the relevant peptide. While splenic CD11b^+^ from the different groups were equally suppressive, CD11b^+^ cells from the tumor of mice treated with CCR1 and CCR5 shRNAs showed a reduced suppressive activity (figure 4A).

To evaluate myeloid cell tumoricidal activity, we co-cultured CD11b^+^ cells from treated mice with 4T1-luciferase cells and enumerated neoplastic cells 18 hours later by luciferase assay (figure 4B). While tumor-infiltrating myeloid cells from control mice did not affect neoplastic cell number, CD11b^+^ cells from CCR1 and CCR5 shRNA treated mice drastically reduced the number of 4T1 cells in culture indicating a tumoricidal action.

Since most of the phenotypic changes in the tumor-infiltrating leukocytes occurred in the polymorphonucleate subset that is also the most prominent population in the 4T1 model, we assessed the effect of Ly6G^+^cell depletion in 4T1 bearing mice undergoing 4PD treatment with shRNAs specific for CCR1 and CCR5 or scrambled shRNAs as control (figure 4C). In mice treated with the isotype control, CCR1 and CCR5 targeted silencing significantly reduced tumor progression compared with the PBS control. Notably, depletion of Ly6G^+^ cells drastically reduced the antitumor activity of CCR1 and CCR5 shRNAs. Conversely, in mice treated with scrambled shRNAs, Ly6G depletion reduced tumor progression whereas we observed no differences from the PBS control in the mice treated with the isotype antibody (figure 4C).

Finally, we used NSG mice to evaluate the possible contribution of B, T and natural killer (NK) cells in the observed antitumor effect. Briefly, BALB/c or NSG mice were challenged with the 4T1 tumor orthotopically and treated intravenously with 4PD loaded with scrambled or CCR1 and CCR5 shRNAs 3, 5, 7, 10 and 12 days after challenge. As observed above, CCR1 and CCR5 targeted silencing significantly inhibited tumor growth in BALB/c mice (figure 4D). A similar antitumor effect was observed also in the NSG mice indicating that T, B, and NK cells have minimal or no effect on the antitumor activity mediated by CCR1 and CCR5 targeted silencing.

Taken together, these data indicate that silencing of CCR1 and CCR5 on myeloid cells changes their function from suppressive to antitumoral and highlight the role of neutrophils in the observed antitumor effect.

### CCR1 and CCR5 blockade promotes the differentiation of tumoricidal neutrophils

**Figure 5:**
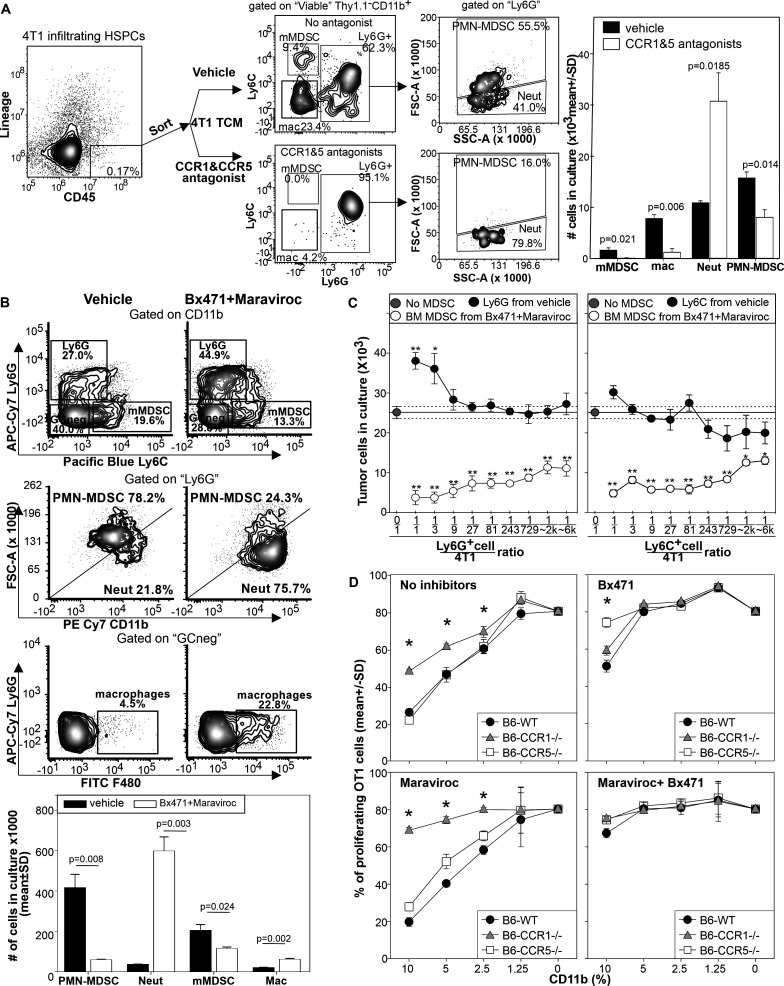
CCR1 and CCR5 regulate HSPC commitment toward MDSCs or tumoricidal neutrophils. (A) Lin-/CD45^+^cells were sorted by FACS from pooled tumors of 14 mice injected 20 days before with 4T1. Sorted cells (5×10^4^) were stimulated by 4T1-TCM in the presence or in the absence of maraviroc and B×471 and enumerated by flow cytometry 4 days later. Data derived from two independent experiments. (B) BALB/c BM cells were cultured with 4T1-TCM and: (i) maraviroc, (ii) B×471, (iii) both antagonists, or (iv) vehicle for 4 days and characterized by flow cytometry. (C) FACS sorted CD11b^+^Ly6G^+^ or CD11b^+^Ly6C^+^cells from the cultures were incubated with 4T1-luc cells at the indicated ratio. (D) BM cells from naive CCR1^−/−^, CCR5^−/−^ or CCR1^+/+^ CCR5^+/+^ C57BL/6 mice were stimulated by MCA203 TCM with maraviroc, B×471, both antagonists, or vehicle. 4 days later, cells were magnetically purified and tested in suppressive assays against OT1 cells stimulated with the relevant peptide. *p<0.01, **p<0.001. CCR, chemokine receptors; HSPCs, hematopoietic stem and precursors cells; PMN-MDSC, polymorphonuclear myeloid-derived suppressor cell; TCM, tumor conditioned media; WT, wild type.

Since HSPC-like cells are found in the cancer host in the periphery and intratumorally (online supplemental figure 5A), express CCR1 and CCR5 (online supplemental figure 5B), and can be transfected by 4PD (figure 1), we evaluated whether CCR1 and CCR5 blockade can affect the differentiation of HSPC-like cells into MDSCs. We isolated intratumoral CD45^+^ Lineage negative hematopoietic precursors from 4T1 bearing mice and cultured them with 4T1 TCM in the presence or absence of the CCR1 and CCR5 antagonists B×471 and maraviroc, respectively (figure 5A). In cultures treated with vehicle, TCM induced the differentiation of macrophages, mMDSCs, FSC-A^high^ PMN-MDSCs, and few classical FSC-A^low^ neutrophils supporting the notion that intratumoral HSPC-like cells can differentiate into more mature myeloid cells. Simultaneous blockade of CCR1 and CCR5 inhibited macrophages, mMDSCs, and PMN-MDSCs differentiation and promoted the differentiation of ‘classical’ FSC-A^low^ neutrophils. To understand if CCR1 and CCR5 blockade modulates not only the phenotype but also the function of myeloid cells induced by tumor derived factors, we employed bone marrow cells from naïve mice. We cultured BM cells with 4T1 TCM with or without CCR1 and CCR5 antagonists (figure 5B) for 4 days and then characterized the resulting populations by flow cytometry. As expected, in the absence of the antagonists, TCM differentiated BM cells largely in CD11b^int^ FSCA^high^ Ly6G^+^PMN-MDSCs, CD11b^high^ Ly6C^+^ mMDSCs, with few contaminations of Ly6G^−^Ly6C^−^F4/80^+^ macrophages and CD11b^high^ FSC^low^ Ly6G^+^neutrophils. Conversely, in the presence of maraviroc and B×471 TCM induced the differentiation of large number of classical neutrophils, increased the number of macrophages, and drastically reduced both mMDSCs and PMN-MDSCs (figure 5B). We then cultured FACS sorted Ly6G^+^ or Ly6C^+^ cells differentiated in the presence or absence of CCR1 and CCR5 antagonists with 4T1-luciferase cells (figure 5C). Compared with the 4T1 seeded alone, the addition of Ly6G^+^cells from the control cultures induced a higher recovery of 4T1 cells supporting the notion that PMN-MDSCs promote neoplastic cell proliferation. In sharp contrast, Ly6G^+^ cells differentiated with maraviroc and B×471 dramatically decreased 4T1 recovery suggesting a strong and direct tumoricidal action. Similar results were observed with Ly6C^+^cells (figure 5C). To further test the involvement of CCR1 and CCR5 in MDSC differentiation, we employed bone marrow from CCR1^−/−^, CCR5^−/−^, or wild type (WT) C57BL/6 mice (figure 5D). As expected, MCA203-TCM drove BM cells from WT mice to differentiate into highly suppressive MDSCs. We observed a slight reduction in MDSC suppressive activity when CCR1^−/−^BM cells were differentiated with no antagonists, whereas no differences in suppressive activity were evident with CD11b^+^cells differentiated from CCR5^−/−^BM. Addition of the CCR1 antagonist B×471 during differentiation had low effect on CCR1^−/−^BM-derived CD11b^+^ cells, it partially reversed the suppressive activity of WT-BM derived myeloid cells, and completely reversed the suppressive activity of CCR5 deficient myeloid cells. Conversely, maraviroc during MDSC differentiation reversed the suppressive activity of myeloid cells from CCR1 deficient BM-cells but had no effect on MDSCs differentiated from the BM of WT or CCR5^–/–^ mice. The combined use of both B×471 and maraviroc during differentiation completely inhibited the suppressive activity of myeloid cells differentiated from all the genetic backgrounds.

Neutrophils have been proposed to exert their tumoricidal action via degranulation and or secretion of nitric oxide, lysozyme, superoxide or neutrophil extracellular traps (NETs).^23–26^ To explore if any of these pathways was involved in neutrophil’s tumoricidal action we performed tumor co-culture assays in the presence of 4,4′-Diisothiocyanatostilbene-2,2′-disulfonic acid disodium salt to inhibit NETosis and degranulation, N, N′, N′′-Triacetylchitotriose to inhibit lysozyme, recombinant superoxide dismutase to neutralize superoxide, imidazole to inhibit the respiratory burst, or N^G^-Methyl-L-arginine to inhibit nitric oxide synthase 2. None of the tested inhibitors reversed neutrophil tumoricidal activity (online supplemental figure 6).

These results support the notion that CCR1 and CCR5 silencing modulates tumor driven differentiation of myeloid precursors by inhibiting MDSC polarization and increasing the number of myeloid cells that induce tumor death by a yet undisclosed mechanism.

### CCR1 and CCR5 signaling mediates the protumoral changes induced in HSPCs by tumor derived factors

**Figure 6:**
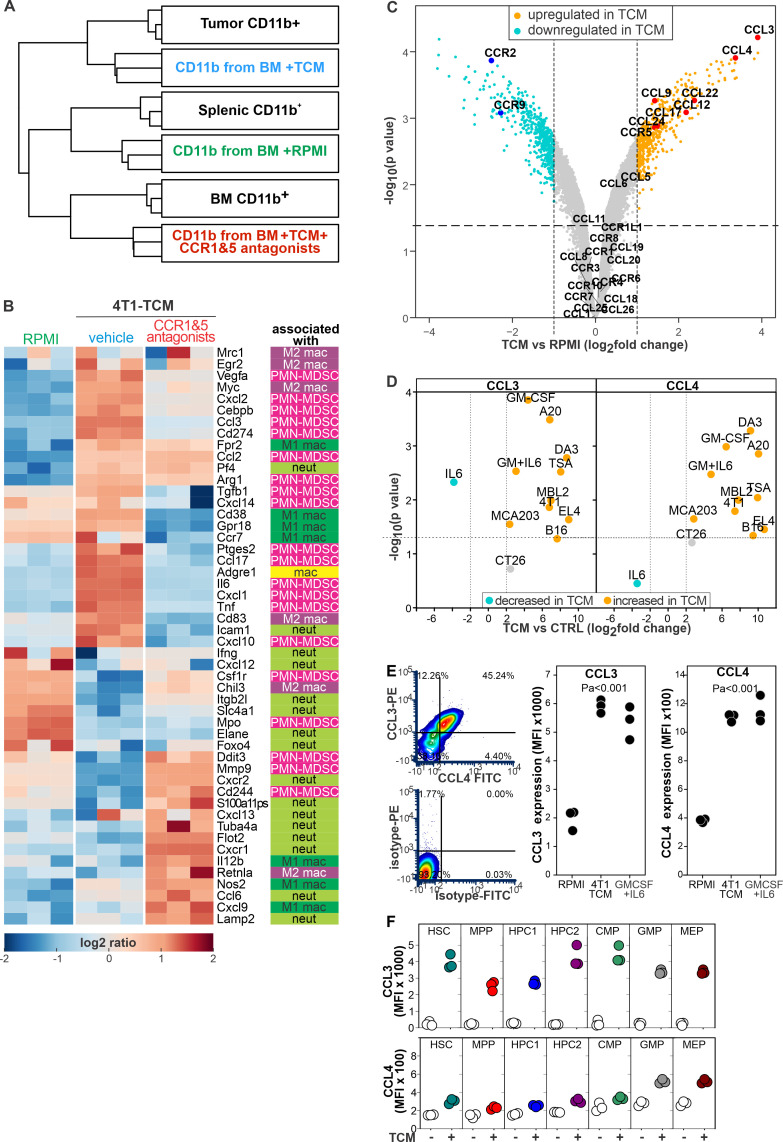
Tumor derived factors induce MDSC commitment via secretion of CCL3 and CCL4 and CCR1 and CCR5 signaling. BALB/c BM cells were cultured in RPMI or 4T1-TCM supplemented with B×471 and maraviroc or with vehicle. Twenty-four hours later, CD11b^+^cells were magnetically isolated, RNA extracted and hybridized on Affymetrix microarrays. (A) Gene expression data of CD11b^+^cells were merged with publicly available transcriptional profiles of CD11b^+^cells isolated from 4T1 tumors or from the BM (BM CD11b^+^) or the spleen (splenic CD11b^+^) of naïve BALB/c mice. Unsupervised clustering of experimental groups based on gene expression profiles. (B) Expression levels of genes associated with neutrophils, PMN-MDSCs, or M1 or M2 macrophages (online supplemental table 3). (C) Volcano plot from the comparison between BM cells stimulated with RPMI or 4T1 TCM. CCRs and CCL are highlighted. (D) CCL3 and CCL4 concentration was evaluated by CBA in the supernatant of BM cells stimulated for 24 hours by the indicated TCMs or recombinant cytokines. The same TCMs incubated for 24 hours without BM cells were used as control. Supernatant from BM cells cultured in RPMI with no stimuli was used as negative control for the cultures with recombinant cytokines. Data derived from two independent experiments. (E) CCL3, CCL4 expression was evaluated by ICS on Lin^-^population of BALB/c BM cells treated for 4 hours with 4T1-TCM, GM-CSF and interleukin 6, or RPMI. (F) BM from C57BL/6 mice was cultured for 4 hour with MCA203 TCM and evaluated by flow cytometry on HSPC subsets. CCR, chemokine receptors; HSPCs, hematopoietic stem and precursors cells; MDSC, myeloid-derived suppressor cell; MPP, multipotent progenitors; TCM, tumor conditioned media.

Because of the absence of suitable markers that allow the easy discrimination of PMN-MDSCs from ‘classic’ neutrophils, we employed transcriptome analysis to determine the phenotype of myeloid cells differentiated by TCM with or without CCR1 and CCR5 antagonists. We cultured BM cells for 24 hours in RPMI media (RPMI) or in TCM in the presence or absence of B×471 and maraviroc (TCM+CCR antagonists). We hybridized the RNA isolated from sorted CD11b^+^ cells on gene expression microarrays and we merged gene expression profiles with publicly available transcriptional data of CD11b^+^ cells from 4T1 tumors and from BM (BM CD11b^+^) and spleen (splenic CD11b^+^) of naïve BALB/c mice (figure 6 and online supplemental table 2). Next, we performed an unsupervised hierarchical clustering analysis of gene expression profiles. BM cells incubated with 4T1-TCM grouped with tumor-infiltrating myeloid cells, BM cells cultured in RPMI media clustered with splenic CD11b^+^ cells, and BM cells cultured with TCM and CCR1 and CCR5 antagonists associated with CD11b^+^ cells from the BM of naïve mice (figure 6A). Next, we investigated the effect of CCR1 and CCR5 antagonists on the transcriptional levels of genes previously associated with PMN-MDSCs, with M1 or M2 macrophages, or with classical neutrophils (figure 6B and online supplemental table 3). As expected, compared with RPMI, TCM upregulated several genes associated to the PMN-MDSC phenotype, suppressive activity, or differentiation (PMN-MDSC, red in figure 6B), and/or a M2 macrophages (purple in figure 6B). Conversely, the CCR1 and CCR5 blockade inhibited the expression of most of these genes and promoted the upregulation of genes associated with classical neutrophils (Neut, light green figure 6B) or M1 macrophages (dark green figure 6B) including lactostranferrin, a glycoprotein with known antitumoral activity.^27^ Functional enrichment analysis showed that B×471 and maraviroc inhibited the activation of metabolic (eg, oxidative phosphorylation response, hypoxia) and signaling pathways (eg, interleukin (IL)6-STAT3, mTORC1) associated with MDSCs and induced by TCM in BM cells (online supplemental figure 7).

10.1136/jitc-2021-003131.supp2 Supplementary data

Since MDSCs can be differentiated in the absence of exogenous CCR1 and CCR5 ligands (eg, by using GM-CSF and IL6),^28^ we evaluated whether TCM upregulates CCR1 and CCR5 ligands. This analysis revealed that the CCR1 and CCR5 ligands CCL3 and CCL4 were among the most significantly upregulated genes (figure 6C).

We next evaluated whether CCL3 and CCL4 induction was a widespread phenomenon or whether it was only associated to the 4T1 tumor. To this aim, we measured CCL2, CCL3, CCL4, and CCL5 by CBA in TCM from different cancer types or in cultures of BM cells stimulated with the same TCMs or with recombinant cytokines involved in MDSC differentiation (figure 6D and online supplemental figure 8). At baseline, we detected elevated concentrations of CCL2 and CCL5 but low concentrations of CCL3 and CCL4 in most TCMs (online supplemental figure 8A). After 24 hours with TCM stimulation or recombinant cytokines involved in MDSC differentiation, BM cultures significantly increased concentrations of CCL3 and CCL4 (range 5–490 and 7–1648 folds for CCL3 and CCL4, respectively) (figure 6D). Recombinant GM-CSF, secreted by most mouse and human tumors,^29^ stimulated both CCL3 and CCL4 (figure 6D). TCM stimulation or any recombinant cytokine tested did not modulate CCL2 or CCL5 (online supplemental figure 8B). Intracellular staining confirmed that almost half of HSPCs secrete CCL3 and CCL4 on stimulation with 4T1 TCM or GM-CSF +IL6 a combination commonly used to differentiate MDSCs (figure 6E).^28^ A similar analysis performed on BM cells from C57BL/6 mice showed that almost all HSPC subsets secreted these chemokines in response to MCA203 TCM (figure 6F and online supplemental figure 8C). Interestingly, the TCM from the poorly immunogenic tumor CT26 and recombinant IL6 alone did not modulate CCL3 or CCL4 production (figure 6D). It is important to remember that IL6 has a dual and opposite actions on the immune response^30^ that are most likely regulated by the signal integration with other factors. For example, GM-CSF and IL6 induced BM cells to differentiate in highly suppressive MDSCs^28^ that promoted tumor cell proliferation and did not induce clonotypic T cell proliferation when pulsed with the relevant peptide (online supplemental figure 8D–F). In sharp contrast, BM cells differentiated with only IL6 were not suppressive, did not increase neoplastic cell proliferation, induced clonotypic T cell proliferation when pulsed with the relevant peptide, and contained a large proportion of MHC class II^+^, CD86^+^ M1-like macrophages (online supplemental figure 8C–G).

Taken together, these data support the notion that CCR1 and CCR5 regulate the differentiation commitment of myeloid cells through a mechanism that involve the autocrine secretion of CCL3 and CCL4 in response to MDSC inducing factors secreted by neoplastic cells.

### CCR1 and CCR5 blockade restrains the generation of human MDSCs

Having shown that CCR1 and CCR5 are required for tumor driven MDSC differentiation in mice, we next evaluated whether a similar pathway regulated tumor induced myelopoiesis in human.

**Figure 7:**
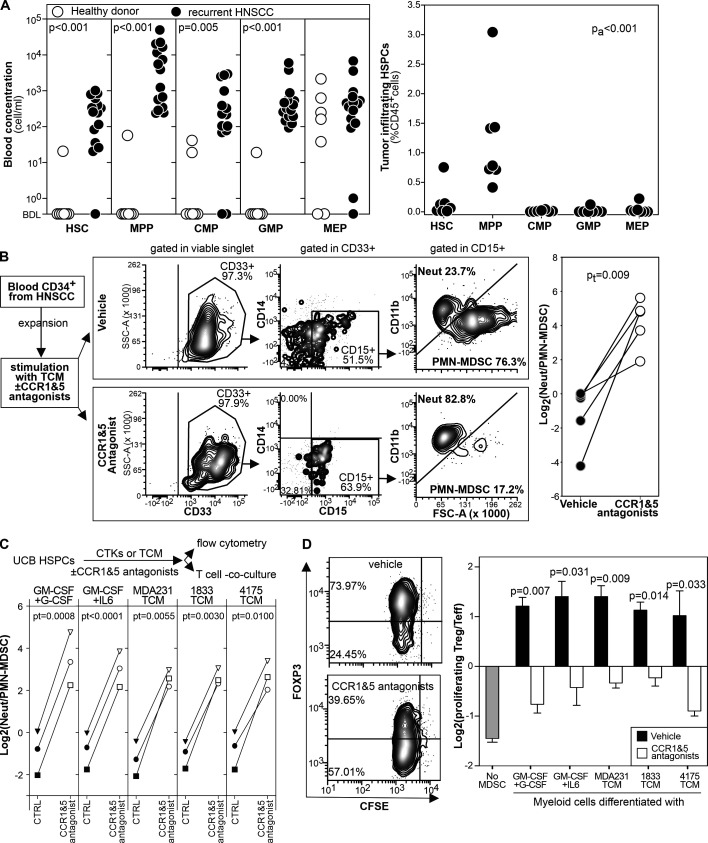
CCR1 and CCR5 antagonists inhibit MDSC differentiation from human HSPCs. (A) The indicated HSPC subsets were enumerated by flow cytometry in the blood and in the tumor of patients with recurrent HNSCC or in age matched healthy controls. (B) Magnetically isolated circulating HSPCs from patients with HNSCC were expanded, stimulated for 4 days with TCM in the presence or in the absence of B×471 and maraviroc, and analyzed by flow cytometry. (C) CD3-depleted, ficolled UCB cells were stimulated for 4 days with cytokines or TCM in the presence or in the absence of maraviroc and B×471. Differentiated cells were then analyzed phenotypically by flow cytometry or in T cell co-culture assays. Neutrophils/PMN-MDSCs ratio of UCB cultures under the indicated conditions are reported. (D) UCB cells differentiated as in (C) were co-cultured with PHA-stimulated autologous, CFSE labeled CD3^+^cells for 3 days and analyzed by flow cytometry. Log_2–_ratio between Foxp3^+^ ‘Treg’ and Foxp3^–^ T effector cells among the CFSE^low^ CD3^+^ CD4^+^ cells. Data derived from 2,3 biological replica. CCR, chemokine receptors; HNSCC, head and neck squamous cell carcinoma; HSPCs, hematopoietic stem and precursors cells; MDSC, myeloid-derived suppressor cell; PHA, phytohemagglutinin; PMN, polymorphonuclear; TCM, tumor conditioned media; UCB, umbilical cord blood.

First, we analyzed by flow cytometry the blood and the tumor of patients with recurrent HNSCC or age matched healthy donors to characterize HSPC subsets (online supplemental figure 9). In the blood, we observed the expansion of cells resembling HSC, MPP, CMP, and GMP whereas we found no significant differences in MEP (figure 7A). As observed in mice, at the tumor site MPP-like cells were the most prominent population (figure 7A).

Next, we evaluated whether circulating HSPC-like cells could differentiate into PMN-MDSC-like cells on stimulation with TCM and whether CCR1 and CCR5 antagonists could alter this process. We purified and expanded CD34^+^ cells from patients with HNSCC and cultured them with TCM with or without B×471 and maraviroc (figure 7B). TCM differentiated HSPC-like cells mostly into CD15^+^ FSC^high^ CD11b^low^ PMN-MDSCs with only a few CD14^+^ monocyte-like and CD15^+^ FSC^low^ CD11b^high^neutrophils. In sharp contrast, HSPCs cultured with TCM, B×471, and maraviroc differentiated into CD15^+^ CD11b^high^ FSC^low^ neutrophils with almost no contamination of PMN-MDSCs and monocytes (figure 7B).

Finally, to functionally evaluate the role of CCR1 and CCR5 on human MDSC differentiation, we stimulated UCB-HSPCs with TCMs from human breast cancer cell lines (MDA231, 1833, or 4175) or recombinant cytokines (GM-CSF +G CSF or GM-CSF +IL6) for 4 days with B×471 and maraviroc (figure 7C) or with vehicle as control. In the vehicle group, PMN-MDSCs outnumbered the FSCs^low^ CD11b^high^ neutrophils regardless of the differentiation conditions whereas ‘classical’ neutrophils outnumbered PMN-MDSCs in the B×471 +maraviroc cultures (figure 7C). When these cells were functionally tested in co-culture experiments with autologous, phytohemagglutinin stimulated CFSE labeled T cells, myeloid cells from the vehicle group induced Foxp3 in most of proliferating T cells^31^ whereas those from the B×471 +maraviroc cultures expanded mostly Foxp3^-^T cells (figure 7D). These results, consistent across all the MDSC inducing conditions tested, indicated that CCR1 and CCR5 mediate tumor-induced MDSC differentiation in humans.

## Discussion

Cancer induced ‘emergency’ hematopoiesis is responsible for the accumulation of protumoral myeloid cells systemically and in the tumor bed, although both protumor PMN-MDSCs and antitumor neutrophils coexist in cancer hosts.^21 32–34^ Despite the importance of PMN polarization in cancer host, the molecular pathways that regulate this protumoral process are still not fully understood.

Using an agnostic nanoparticle-based strategy, we first discovered that the silencing of both CCR1 and CCR5 was necessary and sufficient to change the phenotype and function of tumor-infiltrating myeloid cells and restrain tumor progression in all mouse cancer types tested. Importantly, inhibition of both receptors was required to achieve the therapeutic effect, suggesting redundancy, synergy, or integration of the downstream signaling. Phenotypic and functional analysis of the tumor microenvironment pointed us to the presence of classical neutrophils as the main mechanism for the antitumor activity induced by CCR1 and CCR5 silencing. Indeed, we did not observe any changes in the phenotype or number of tumor-infiltrating lymphocytes in mice treated with CCR1 and CCR5 shRNAs but we observed a modification of phenotype and function of PMN-cells, macrophages, and DCs. Additionally, treatment with anti-Ly6G antibody further implicates neutrophil polarization as primarily responsible for the observed antitumor activity. This antibody that depletes PMN but leaves untouched T and NK subsets significantly reduced the therapeutic effects of CCR1 and CCR5 shRNAs but inhibited tumor progression in mice treated with the scrambled shRNAs. A marginal role of T, B and NK cells in the observed antitumor effect is confirmed by our experiment in NSG mice that are devoid to these lymphoid populations. Although these results do not exclude a possible tardive role of the adaptive immunity in mediating cancer control, they do indicate that the relative concentration of PMN-MDSCs and classical neutrophils is key for the observed antitumor effects.

‘Classical’ neutrophils can exert a strong tumoricidal action directly and in a contact dependent manner by secreting different factors (eg, nitric oxide, hydrogen peroxide, superoxide, lactotransferrin, NETs) and via trogoptosis, or indirectly by promoting adaptive immunity.^32 35–37^ In vitro assays using commercially available inhibitors seem to deny the involvement of nitric oxide, lysozyme, superoxide, and NETosis in the tumoricidal action of our ‘converted’ neutrophils. Although beyond the scope of this manuscript, that focuses on myeloid cell repolarization, the upregulation of lactotransferrin by CCR1 and CCR5 antagonists points to this glycoprotein as putative mediator of neutrophil cytotoxicity.^27^

While most of our data are on neutrophils and PMN-MDSCs, since these are the most abundant populations found in most human and mouse tumors,^38 39^ including the models evaluated, we also observed a polarization of macrophages toward an M1-like phenotype in the tumors of mice treated with shRNAs specific for CCR1 and CCR5, and a loss of suppressive activity of Ly6C^+^mMDSC differentiated in vitro by tumor derived factors in the presence of CCR1 and CCR5 inhibitors. These effects are not completely surprising since different transcription factors modulated by CCR1 and CCR5,^40–42^ such as C/EBPβ or STAT3, play important roles in the polarization of multiple myeloid cell subsets including MDSCs, macrophages, and DCs.^28 43–46^ This can also explain why the targeted silencing of CCR1 and CCR5 affects tumor growth across multiple models even when PMN-MDSCs are not the most abundant protumoral myeloid cells like, for example, the MCA203 model.

CCR1 and CCR5 are detectable in most human tumors and are usually associated with leukocyte trafficking.^47^ Genetic data, however, indicated a modest chemotactic effect of CCR1 and CCR5 in myeloid cell trafficking and showed the dominant role of CCR2 in this process.^12^ Our adoptive cell transfer experiment confirmed these findings and argued against a prominent role of CCR1 and CCR5 in myeloid cell trafficking to the tumor. Instead, we found that the simultaneous inhibition of CCR1 and CCR5 prevents MDSC differentiation driven by tumor derived factors or recombinant cytokines and promotes the generation of neutrophils with a strong antitumor activity. We confirmed these findings in mouse and human not only using HSPCs from naïve mice or from human umbilical cord blood but also using circulating HSPC-like cells from tumor bearing mice and patients with HNSCC. These data indicate that CCR1 and CCR5 play an unexpected role in myeloid cell polarization even if HSPCs-like have been previously sensitized by growing tumors. It is important to note that this is not the first time that CCR1 and CCR5 have been associated with chemotaxis independent functions. For example, both receptors mediate osteoclast differentiation and function and directly enhance cancer cell survival and proliferation, and CCR5 seems to be implicated in MDSC suppression.^48 49^

Our transcriptome analysis further confirmed the involvement of CCR1 and CCR5 in the polarization of myeloid cells, showing that HSPCs differentiated by tumor derived factors in the presence of maraviroc and B×471 shared a signature of classical neutrophils and clustered with CD11b^+^ cells from the bone marrow of naïve mice, whereas the ones differentiated without antagonists showed an MDSC signature and clustered with tumor-infiltrating myeloid cells.

The transcriptome analysis also revealed that tumor derived factors induced bone marrow cells to transcribe CCL3, CCL4, and other ligands of CCR1 and CCR5. CCL3 and CCL4 are produced by different leukocyte populations and their presence in human malignancies is associated with the presence of protumoral myeloid cells and worse prognosis in mouse models^50 51^ and in patients with cancer.^12 52–54^

This autocrine production of CCL3 and CCL4 by HSC, confirmed at the protein level by cytokine beads array and intracellular staining, explained the conundrum that MDSCs can be differentiated in the absence of CCR1 and CCR5 ligands using recombinant cytokines such GM-CSF and IL6 or tumor conditioned media deficient of CCL3 and CCL4. Interestingly, while GM-CSF increases the secretion of both chemokines, IL6, that is co-required for MDSC generation,^28^ significantly decreases CCL3 and does not modulate CCL4. IL6 is involved in HSCs expansion and in emergency hematopoiesis^55 56^ and has been describe to induce both immune stimulatory and immunosuppressive myeloid cells in different experimental settings.^30^ These opposite functions can be explained by the integration of IL6 signaling with the one of other of factors present in the tumor micro-environment and macro-environment. For example, our data confirmed that GM-CSF and IL6 induces highly suppressive MDSC^28^ and showed that this combination upregulates CCL3 and CCL4. In sharp contrast, IL6 alone do not differentiate BM cell in MDSC, do not upregulate CCL3 and CCL4, and promote the differentiation of cells capable to stimulate T cell proliferation.

In summary, our data support a model by which cancer-driven protumoral myelopoiesis is regulated by a circuit in which tumor derived factors induce the production of CCL3 and CCL4 and other CCR1 and CCR5 ligands that, by engaging CCR1 and CCR5, autocrinally promote their differentiation into MDSCs.

10.1136/jitc-2021-003131.supp3 Supplementary data

## Data Availability

Data are available in a public, open access repository. Data are available upon reasonable request. All data relevant to the study are included in the article or uploaded as supplementary information. Raw data are available at Gene Expression Omnibus under accession number GSE148615.
