# Supplementary material for: CCR1 and CCR5 mediate cancer-induced myelopoiesis and differentiation of myeloid cells in the tumor
Source: J Immunother Cancer. 2022 Jan 21;10(1):e003131. doi: 10.1136/jitc-2021-003131 (PMC8785210; doi:10.1136/jitc-2021-003131)
Supplement: Supplementary data [file jitc-2021-003131supp001.pdf]

Supplemental methods

Flow Cytometry

Flow cytometry was performed on a daily-calibrated flow cytometer, using titrated and validated antibody controls, vital dye, automatic compensation using single-color staining, and FMO or isotype antibodies as negative controls. Antibodies used are indicated in supplemental table 2. Purified CCL4 antibody were conjugated with AlexaFluor™488 Antibody Labeling Kit and used with the BD Cytofix/Cytoperm™Plus kit following manufactured instructions. Mouse and human FoxP3 intracellular staining were performed using eBioscience™ Foxp3/Staining Buffer. Dead cells were excluded by the analysis by using LIVE⁄DEAD® Stain. Samples were read on a LSR2 (BD Bioscience) equipped with 405nm, 488nm, 532nm, and 635nm lasers or Cytoflex (Beckman Coulter) equipped with 405nm, 488nm, 561nm, and 638nm lasers. Sortings were performed on the MoFlo Astrios cell sorter (Beckman Coulter). Data were analyzed using FCSexpress-plus vs7(Denovo-Software).

Image Cytometry

4 µm thick sections from FFPE tumors underwent deparaffination and antigen retrieval with TRIS-EDTA (pH=9). Section were then incubated with Image-iT™FX-Signal-Enhancer (Invitrogen) (30' @RT), with blocking solution (1h @RT, PBS 1x/Triton-x100 0,02%/FBS 10%) and with the purified rabbit monoclonal-anti-Rb primary antibody (Abcam, clone:EPR17512) and purified rat monoclonal anti-mouse anti-LAMP-2 (CD107b) antibody (BioLegend, clone M3/84) (ON @ 4ºC). Sections were washed and incubated 2hs @RT with donkey anti-Rabbit AF647 AffiniPure (Jackson Labs) and donkey anti-Rat AF555 (Invitrogen), section were washed and then incubated 1.5 hs with AF488-conjugated rat anti-mouse Ly6G (BioLegend, clone:1A8). Stained slides were scanned at 20X with the VS120 microscope (Olympus) using DAPI, FITC and TRITC and Cy5 cubes. Tiff files were processed with cellprofiler (www.cellprofiler.com, Supplemental Fig.3) and fed into FCSexpress-plus vs7(Denovo software).

Quantitative RT-PCR

Trizol extracted RNA was retrotranscribed with the High-Capacity-cDNA-Reverse-Transcription Kit and amplified using TaqMan probes and the TaqMan-Fast-Universal-PCR-Master-Mix kit on the StepOnePlus™thermocycler.

Cytokine Beads Array

CCL2, CCL3, CCL4, and CCL5 were quantified using a custom MILLIPLEX® Multiplex Assays (Millipore-Sigma) following manufacturer instruction.

Cell isolation

Human $CD3^+$ and $CD3^-$ cells from UCB were isolated using the Pan T Cell Isolation Kit, human (Miltenyi Biotec). *Mouse $CD11b^+$cells* have been magnetically isolated from tumor and/or spleen of tumor bearing mice using the CD11b Microbeads, human and mouse kit (Miltenyi Biotec) according to manufacturer instruction.

Mouse tumor infiltrating HSPCs have been isolated from a pool of fourteen 4T1 tumors. Tumor single cell suspensions were depleted of Lineage positive cells using the Direct Lineage Cell Depletion Kit, mouse (Miltenyi Biotec) following manufacturer instruction, stained with Anti-Mouse Lineage cocktail and anti-CD45 (Biolegend), and sorted by FACS as viable $CD45^+/Lin^-$ cells.

Human $CD34^+$cells from the blood of HNSCC patients were isolated from PBMCs of patients with recurrent HNSCC using the diamond $CD34^+$isolation kit (Miltenyi Biotec) following manufacturer's instructions.

MDSC differentiation and Functional assays

MDSC differentiation. $6x10^5$ BM cells were cultured in 30% TCM or complete media with either rmGM-CSF and rmIL6, rmGM-CSF and rmG-CSF, or rmIL6 alone (40 ng/mL each) in low adherence 24 well plate for 4 days. Maraviroc (10 µM) and/or Bx471 (5 µM) were added on day 0 and 3 when indicated.

Mouse MDSCs differentiation from sorted tumor infiltrating HSPCs. HSPCs have been differentiated into MDSCs using the 4T1 TCM as previously above, in presence of CCR1 and CCR5 inhibitors or relative vehicle.

Human-MDSC differentiation from UCB. Ficolled purified, RBC lysed UCB were magnetically depleted by $CD3^+$cells and 2×10^6 cells/well cultured for 4 days in 24-well ultra-low attachment plates (Corning) in RPMI 15% FBS media with rhuGM-CSF and rhuG-CSF, rhuGM-CSF and rhuIL6, or with tumor conditioned media from MDA-MB231, 1833 or 4175.

Expansion of circulating HSPCs from HNSCC and MDSC differentiation. $CD34^+$cells, purified from PBMCs of patients with recurrent HNSCC using the Miltenyi diamond $CD34^+$isolation kit following manufacturer's instructions, (50,000 cells/mL) were expanded in U-bottom 96 well plate in 200 µL of StemSpan™ SFEM media (Stemcells technology) supplemented with stem cell factor (100 ng/mL), FLT3 (100 ng/mL), thrombopoietin (100 ng/mL) IL3 (20 ng/mL) for 1-2 weeks and maintained at a concentration of $\sim2.5x10^5$/mL by dilution with cytokine containing expansion media every 3-4 days. Cells were then washed twice and $2.5x10^5$ cells plated in 1 mL of TCM or complete media in 24 well plate.

Mouse-MDSCs suppressive assay. 10^5 CFSE labeled splenocytes from Cl4 mice or OT1 were stimulated with

the relevant peptide (1 µM) in the presence of 10^6 syngeneic splenocytes and syngeneic CD11b$^+$cells for 3 days in 96 well flat bottom plates. Proliferation was evaluated by flow cytometry on the viable CD3$^+$CD8$^+$population.

Human-MDSCs suppressive assay. 10^5 magnetically purified, CFSE labeled, CD3$^+$T cells from UCB were stimulated by PHA (6%) in AIM-V media (Gibco) for 3 days in 96-well U-bottom plates in the presence or absence of 5×10^4 UCB-derived autologous MDSC cells. HEK-293 cells were used as non-suppressive control.

Tumor-Myeloid cell co-culture assay. Magnetically isolated CD11b$^+$cells (purity >90% by flow cytometry) or FACS sorted Ly6G or Ly6C cells were cultured at different ratio with $0.2\text{x}10^5$ 4T1-luciferase cells, in complete medium for 18h at 37°C. 4T1-luciferase cells were enumerated using the Li-COR system by luciferase assay after a 5' incubation with luciferin at 37ºC using a freshly diluted known number of 4T1-luciferase cells as standard curve. Inhibitors targeting the main neutrophil tumoricidal pathway were chosen through a literature search, used at optimal reported concentration, and added to the cultures.

Analysis of microarray gene expression data

Twenty-four hours after culture with RPMI, 4T1 TCM with Bx471 and Maraviroc, or 4T1 TCM with vehicle, BM cells were washed with PBS and RNA extracted by Trizol (Invitrogen) and cleaned with RNeasy columns (Qiagen). For each chip, 2.5 µg of total RNA was amplified to biotinylated complementary RNA (cRNA) as described in the Affymetrix GeneChip® Expression Analysis Technical Manual. Pre-hybridization quality controls were performed with the Agilent 2100 bioanalyzer (Agilent Technologies). RNA from 3 biological replicates was then hybridized on Affymetrix MG-U74Av2 arrays. Microarray probe fluorescence signals were converted to $\log_2$ expression values using the Robust Multiarray Average procedure of the *affy* Bioconductor package in R. Briefly, fluorescence intensities were background-adjusted and normalized using quantile normalization, and expression values were calculated using median polish summarization and a custom chip definition file for the Mouse Gene 2.0 ST array based on Entrez genes (mogene20st_Mm_ENTREZG version 21.0.0). Raw data are available at Gene Expression Omnibus under accession number GSE148615.

To identify differentially expressed genes, we compared the expression levels of BM cells cultured in 4T1-TCM and in complete media (RPMI) using the Significance Analysis of Microarray (SAM) algorithm coded in the *samr* R package. [1] In SAM, we estimated the percentage of false positive predictions (i.e. False Discovery Rate, FDR) with 100 permutations and selected those gene IDs with FDR ≤ 5% and absolute fold change larger than a selected threshold (e.g. ≥ 2). The volcano plot, showing the most significantly differentially expressed genes in

the comparison of BM CD11b$^+$cells from 4T1-TCM and complete media (RPMI), was generated using the *ggplot* function of the *ggplot2* R package. P-values were derived from SAM q-values using the function *samr.pvalues.from.perms* of the *samr* R package.

The gene expression levels of CD11b$^+$cells from BM cells cultured with RPMI, 4T1-TCM with vehicle and 4T1-TCM with Bx471 and Maraviroc have been merged with publicly available gene expression data of CD11b$^+$cells. Specifically, gene expression data of CD11b$^+$cells infiltrating 4T1 tumors (MDSC; GSM545536, GSM545537, and GSM545538) and of CD11b$^+$cells isolated from the BM (BM CD11b$^+$; GSM545545, GSM545546, and GSM545547) and from the spleen (splenic CD11b$^+$; GSM545524, GSM545525, and GSM545526) of naïve BALB/c mice were obtained from GSE21927. Raw .CEL files were converted to $\log_2$ expression values using the Robust Multiarray Average procedure of the *affy* Bioconductor package in R a custom chip definition file for the Affymetrix Mouse Genome 430 2.0 arrays based on Entrez genes (Mouse4302_Mm_ENTREZG version 21.0.0). Transcriptional data of the samples hybridized on the different microarray platforms have been merged matching the 17,677 common Entrez Gene IDs of the two custom CDFs. A direct merging of raw fluorescence signals (i.e., of CEL files), although desirable for an optimal removal of batch effects, was unfeasible due to the different probe sequences synthesized on the two types of microarrays. Consequently, batch effects have been removed applying the *ComBat* function of the *sva* Bioconductor package to the merged matrix. *ComBat* was used with default parameters.

Unsupervised and supervised clustering were performed using the function *hclust* of R stats package with Pearson correlation as distance metric and average agglomeration method. Before unsupervised clustering, to reduce the effect of noise from non-varying genes, we removed those Entrez Gene IDs with a coefficient of variation smaller than the median of the coefficients of variation in the entire dataset. Gene expression heatmaps have been generated using the function *heatmap.2* of R gplots package after row-wise standardization of the expression values.

Over-representation analysis was performed using Gene Set Enrichment Analysis and gene sets derived from the Hallmark and BioCarta pathway collections of the Molecular Signatures Database (MSigDB; https://www.gsea-msigdb.org/gsea/msigdb). The GSEA software (http://www.broadinstitute.org/gsea/index.jsp) was applied on log2 expression data of BM cells cultured in 4T1-TCM without CCR1 and CCR5 antagonists (4T1-TCM vehicle), in 4T1-TCM supplemented with CCR1 and CCR5 antagonists (4T1-TCM+antagonists), and

in complete media (RPMI). Gene sets were considered significantly enriched at FDR <5% when using Signal2Noise as metric and 1,000 permutations of gene sets. Except for the over-representation analysis, all data analyses were performed in R version 3.5.1.

Supplemental table 1: material and reagents

REAGENT or RESOURCE	SOURCE	IDENTIFIER
**Antibodies**		
Rat anti-Mouse CCL3	ThermoFisher Scientific	Cat# 12-7532-82, RRID:AB_2572662
Goat anti-Mouse CCL4	Abcam	Cat# ab10386, RRID:AB_2071053
Rat anti-Mouse CCR1	BioLegend	Cat# 152506, RRID: AB_2687211
Hamster anti-Mouse CCR5	BioLegend	Cat# FAB1802A, RRID: AB_357091
Hamster anti-Mouse CCR5	BioLegend	Cat# 107006, RRID: AB_313301
Rat anti-Mouse CD11b	BD Biosciences	Cat# 562950, RRID: AB_2737913
Rat anti-Mouse CD11b	BD Biosciences	Cat# 550993, RRID: AB_394002
Rat anti-Mouse CD11b	BD Biosciences	Cat# 563168, RRID:AB_2716860
Hamster Anti-Mouse CD11c	BD Biosciences	Cat# 558079, RRID: AB_647251
Rat anti-Mouse CD150	BioLegend	Cat# 115918, RRID:AB_2239178
Rat anti-Mouse CD150	BioLegend	Cat# 115926, RRID:AB_2562190
Rat anti-Mouse CD117	BD Biosciences	Cat# 553356, RRID:AB_398536
Rat anti-Mouse CD117	BD Biosciences	Cat# 563399, RRID:AB_2738183
Rat anti-Mouse CD16/CD32	BD Biosciences	Cat# 553142 RRID: AB_394657
Rat anti-Mouse CD16/32	BioLegend	Cat# 101333, RRID:AB_2563692
Rat anti-Mouse CD206	BioLegend	Cat# 141723, RRID AB_2562445
Rat anti-Mouse CD206	BioLegend	Cat#141729 RRID: AB_2565823
Rat anti-Mouse CD24	BioLegend	Cat# 101808, RRID: AB_312841
Rat anti-Mouse CD3	ThermoFisher Scientific	Cat# 46-0032-82 RRID: AB_1834427
Hamster anti-Mouse CD34	BioLegend	Cat# 128610, RRID:AB_2074601
Rat anti-Mouse CD4	BD Biosciences	Cat# 553049 RRID: AB_394585
Rat anti-Mouse CD45	BioLegend	Cat# 103154, RRID:AB_2572116
Rat anti-Mouse CD45	BD Biosciences	Cat# 557659, RRID:AB_396774
Hamster Anti-Mouse CD48	BioLegend	Cat# 103422, RRID:AB_2075051
Rat anti-Mouse CD8	BD Biosciences	Cat# 553033, RRID:AB_394571
Rat anti-Mouse CD8	BD Biosciences	Cat# 563046, RRID:AB_2737972
Rat anti-Mouse F4/80	AbD Serotec	Cat# MCA497F, RRID: AB_322047
Rat anti-Mouse FoxP3	ThermoFisher Scientific	Cat# 17-5773-80, RRID: AB_469456
Rat anti-Mouse Ly6G	BD Biosciences	Cat# 560600, RRID:AB_1727561
Rat anti-Mouse Ly6G	BD Biosciences	Cat# 560601, RRID:AB_1727562
Rat anti-Mouse Ly6C	BD Biosciences	Cat# 560594, RRID:AB_1727559
Rat anti-Mouse Ly6C	BD Biosciences	Cat# 553104, RRID:AB_394628
Rat anti-Mouse I-A/I-E	BioLegend	Cat# 107606, RRID: AB_313321
Rat anti-Mouse I-A/I-E	BD Biosciences	Cat# 557000, RRID: AB_396546
Anti-Mouse Lineage cocktail	BioLegend	Cat# 133310, RRID:AB_11150779
Anti-Mouse Lineage cocktail	BioLegend	Cat# 133302, RRID:AB_10697030,
Rat anti-Mouse Sca-1	BioLegend	Cat# 108114, RRID:AB_493596
Mouse anti-Human CD11b	BD Biosciences	Cat# 562723, RRID:AB_2737746
Mouse anti-Human CD123	BioLegend	Cat# 306016, RRID:AB_2264693
Mouse anti-Human CD14	BD Biosciences	Cat# 557831, RRID:AB_396889
Mouse anti-Human CD15	BD Biosciences	Cat# 563142, RRID:AB_2738026
Mouse anti-Human CD3	BD Biosciences	Cat# 552851, RRID:AB_394492
Mouse anti-Human CD33	BD Biosciences	Cat# 555626, RRID:AB_395992
Mouse anti-Human CD34	BioLegend	Cat# 343616, RRID:AB_2629726
Mouse anti-Human CD38	BioLegend	Cat# 303506, RRID:AB_314358
Mouse anti-Human CD4	BD Biosciences	Cat# 558116, RRID:AB_397037
Mouse anti-Human CD45	BioLegend	Cat# 304036, RRID:AB_2561940
Mouse anti-Human CD45R	BioLegend	Cat# 103227, RRID:AB_492876
Mouse anti-Human CD8	BD Biosciences	Cat# 557746, RRID:AB_396852
Mouse anti-Human CD90	BioLegend	Cat# 328114, RRID:AB_893431
Mouse anti-Human FoxP3	ThermoFisher Scientific	Cat# 17-4777-42, RRID:AB_10804651
Anti-Human Lineage cocktail	BioLegend	Cat# 348801, RRID:AB_10612570
Rat anti-Mouse Ly6G	BioLegend	Cat#127626, RRID: AB_2561340
Rat Anti-momuse CD107b (LAMP2)	BioLegend	Cat# 108502, RRID: AB_313383
Rabbit anti-Mouse Rb	Abcam	Cat# ab181616, RRID: N/A
AF488 F(ab')$_2$ Donkey Anti-Rabbit IgG	Jackson ImmunoResearch	Cat# 711-546-152, RRID:AB_2340619

**Biological Samples**		
UCB	New York Blood Center, Long Island City, NY, USA	N/A
Blood from HNSCC patients	University of Miami Hospital, Miami, FL, USA	ClinicalTrials.gov Identifier: NCT02544880
**Chemicals, Peptides, Recombinant Proteins, and cell culture media**		
DAPI (4′,6-diamidino-2-phenylindole)	ThermoFisher Scientific	Cat# D1306, RRID:AB_2629482
eBioscience™ CFSE	ThermoFisher Scientific	Cat# 65-0850-84
CellTrace™ Far Red Cell Proliferation Kit	ThermoFisher Scientific	Cat# C34564
LIVE/DEAD™ Fixable Near-IR Dead Cell Stain Kit	ThermoFisher Scientific	Cat# L34976
LIVE/DEAD™ Fixable Yellow Dead Cell Stain Kit	ThermoFisher Scientific	Cat# L34968
Zombie Violet™ Fixable Viability Kit	BioLegend	Cat# 423114
VivoGlo™ Luciferin, In Vivo Grade	Promega	Cat# P1042
Collagenase IV from Clostridium histolyticum	Millipore Sigma	Cat# C5138-500MG
Animal-Free Recombinant Human IL-6	PeproTech	Cat# AF-200-06
Animal-Free Recombinant Murine IL-6	PeproTech	Cat# AF-216-16
Animal-Free Recombinant Human GM-CSF	PeproTech	Cat# AF-300-03
Animal-Free Recombinant Murine GM-CSF	PeproTech	Cat# AF-315-03
Animal-Free Recombinant Human G-CSF	PeproTech	Cat# AF-300-23
Animal-Free Recombinant Mouse G-CSF	PeproTech	Cat# AF-250-05
Animal-Free Human Flt3 Ligand	PeproTech	Cat# AF-HHSC3
Animal-Free Human SCF	PeproTech	Cat# AF-HHSC3
Animal-Free Human TPO	PeproTech	Cat# AF-HHSC3
Animal-Free Human IL-3	PeproTech	Cat# AF-HHSC3
Animal-Free Human Hematopoietic Stem Cell Expansion Cytokine Package (IL-3)	PeproTech	Cat# AF-HHSC3
Influenza HA(518-526)	AnaSpec	Cat# AS-21158
OVA (257-264)	AnaSpec	Cat# AS-60193-1
PHA Reagent Grade	ThermoFisher Scientific	Cat# R30852701
Maraviroc	Millipore Sigma	PZ0002-5MG, CAS Number 376348-65-1
Bx471	Millipore Sigma	SML0020, CAS Number: 217645-70-0
4PD in vivo MDSC/ Transfection Kit	Kerafast	Cat# EMI007
Cardiolipin sodium salt from bovine heart	Millipore Sigma	Cat# C0563
StemSpan SFEM	Stem Cell Technologies	Cat # 09650
RPMI 1640 Medium, GlutaMAX™ Supplement	ThermoFisher Scientific	Cat# 61870036
Keratinocyte SFM (1X)	ThermoFisher Scientific	Cat# 17005042
AIM-V media	ThermoFisher Scientific	Cat# 12055091
Image-iT™FX-Signal-Enhancer	ThermoFisher Scientific	Cat# I36933
TRIzol™ Reagent	ThermoFisher Scientific	Cat# 15596018
Histopaque®-1077	Millipore Sigma	Cat# 10711
Complete media: RPMI1640	ThermoFisher Scientific	Cat# A1049101
Complete media: Heat inactivated FBS 10%	ThermoFisher Scientific	Cat# 16000044
Complete media: HEPES 10mM	ThermoFisher Scientific	Cat# 15630080
Complete media: Penicillin-Streptomycin (50U/ml)	ThermoFisher Scientific	Cat#15070063
Complete media: L-Glutamine (2 mM)	ThermoFisher Scientific	Cat# 25030081

**Critical Commercial Assays**		
CD11b MicroBeads, human and mouse	Miltenyi Biotec	Cat# 130-049-601
Pan T Cell Isolation Kit, human	Miltenyi Biotec	Cat# 130-096-535
Diamond CD34 Isolation Kit, human	Miltenyi Biotec	Cat# 130-094-531
Direct Lineage Cell Depletion Kit, mouse	Miltenyi Biotec	Cat# 130-110-470
MILLIPLEX MAP Mouse Cytokine/Chemokine Magnetic Bead Panel - Immunology Multiplex Assay	Millipore Sigma	Cat# MCYTOMAG-70K (custom)
Fixation/Permeabilization Solution Kit with BD GolgiPlug™	BD Biosciences	Cat# 555028
eBioscience™ Foxp3/Staining Buffer Set	ThemoFisher Scientific	Cat# 00-5521-00
High-Capacity cDNA Reverse Transcription Kit	Applied Biosystems	Cat# 4368813
TaqMan-Fast Universal PCR Master Mix (2X), no AmpErase™ UNG" kit	Applied Biosystems	Cat# 4367846
mCCR1 Mm00438260_s1	Applied Biosystems	Cat# 4331182
mCCR2 Mm04207877_m1	Applied Biosystems	Cat# 4331182
mCCR5 Mm01963251_s1	Applied Biosystems	Cat# 4331182
mCCR7 Mm01301785_m1	Applied Biosystems	Cat# 4331182
Eukaryotic 18S rRNA Hs99999901_s1	Applied Biosystems	Cat# 4331182
AlexaFluor488 Antibody Labeling Kit	ThermoFisher Scientific	Cat# A20181
Affymetrix Mouse Gene ST 2.0 Array	Applied Biosystems	Cat# 902118
**Deposited Data**		
GeneChip raw data		GSE148615
gene expression data of CD11b⁺cells infiltrating 4T1 tumors		GSM545536, GSM545537, GSM545538
gene expression data of CD11b⁺cells isolated from the BM		GSM545545, GSM545546, GSM545547
gene expression data of CD11b⁺cells from the spleen of Naïve BALB/c		GSM545524, GSM545525, GSM545526
**Experimental Models: Cell Lines**		
4T1HAThy1.1luciferase	provided by I. Borrello (Johns Hopkins University, Baltimore, MD, USA) [2]	N/A
4T1	ATCC® [3]	Cat# CRL-2539, RRID:CVCL_0125RL-2539™
CT26	ATCC® [4]	Cat# CRL-2638, RRID:CVCL_7256
TS/A	provided by V. Bronte (University of Verona, Verona, Italy)[5]	RRID:CVCL_F736
MCA203	provided by V. Bronte (University of Verona, Verona, Italy) [6]	N/A
B16Lu8	provided by V. Bronte (University of Verona, Verona, Italy) [7]	N/A
DA3	provided by D. Lopez (University of Miami, Miami, FL, USA) [8]	RRID:CVCL_5419
B4B8	provided by G. Thomas (University of Miami, Miami, FL, USA) [9]	RRID:CVCL_0B35
MDA-MB231	ATCC® [10]	Cat# HTB-26, RRID:CVCL_0062
MDA-BoM-1833	provided by M. Lippman (University of Miami, Miami, FL, USA) [10]	RRID:CVCL_DP48
MDA231-LM2-4175	provided by M. Lippman (University of Miami, Miami, FL, USA) [10]	RRID:CVCL_5998

**Experimental Models: Organisms/Strains**		
BALB/cJ	The Jackson Laboratory	IMSR Cat# JAX:000651, RRID:IMSR_JAX:000651
C57BL/6J	The Jackson Laboratory	IMSR Cat# JAX:000664, RRID:IMSR_JAX:000664
CCR5KO mice B6.129P2-Ccr5<tm1Kuz>/J,	The Jackson Laboratory	IMSR Cat# JAX:005427, RRID:IMSR_JAX:005427
CBy.PL(B6)-*Thy1*^{a}/ScrJ	The Jackson Laboratory	IMSR Cat# JAX:005443, RRID:IMSR_JAX:005443
Cl.4 mice (Tg(TcraCl4,TcrbCl4)1 Shrm/ShrmJ) BALB/c	Gift from E. Sotomayor	IMSR Cat# JAX:005686, RRID:IMSR_JAX:005686
OT1 C57BL/6-Tg(TcraTcrb)1100Mjb/J	The Jackson Laboratory	IMSR Cat# JAX:003831, RRID:IMSR_JAX:003831
CCR1KO B6.129S-*Ccr1*^{tm1Gao}/AdlJ	Provided by P. Murphy (NIH)	IMSR Cat# JAX:032932, RRID:IMSR_JAX:032932

**Oligonucleotides**		
**name**	**Reference**	**sequence**
mCCR1-m3xRT3	[11] Eurofins genomics	5’TTTGACCTTCTTCTCACTGGGTCTTC3’
mCCR1-m3xs300	[11] Eurofins genomics	5’GCTGTCTCTGATCTGGTCTTCCTT3’
CCR1-m3xs600	[11] Eurofins genomics	5’GAGTTCACTCACCGTACCTGTAGC3’
Neo Cassette ANeo	[11] Eurofins genomics	5’TGGGTGGAGAGGCTTTTTGCTTCCTCTTGC3’
mCCR5-5oIMR0700	https://www.jax.org/Protocol?stockNumber=002782&protocolID=22436	5’CAGGCAACAGAGACTCTTGG3’
Primer for mCCR5 oIMR0701	https://www.jax.org/Protocol?stockNumber=002782&protocolID=22436	5’-TCATGTTCTCCTGTGGATCG-3’
Primer for Neo Cassette oIMR6916	https://www.jax.org/Protocol?stockNumber=002782&protocolID=22436	5’-CTTGGGTGGAGAGGCTATTC-3’
Primer for Neo Cassette oIMR6917	https://www.jax.org/Protocol?stockNumber=002782&protocolID=22436	5’-AGGTGAGATGACAGGAGATC-3’
mCCR1shRNA	[12] Oligofactory	GGfUfCfUAAGAAGAGAAfUGAGfUfUfCAAGAGAfCfUfCAfUfUfCfUfCfUfUfCfUfUAGAfCfCfUfUfUfUfU
mCCR2shRNA	[13] Oligofactory	GfCfUAAAfCGfUfCfUfCfUGfCAAGfUfUfCAAGAGAfUGfCfUAAAfCGfUfCfUfCfUGfCAAAfUfUfUfUfU
mCCR5 shRNA	[14] Oligofactory	GGfUfUfCfUGAGAfUfCfUGGfUAAAGAfUfUfCAAGAGAfUfCfUfUfUAfCfCAGAfUfCfUfCAGAAfUfUfUfUfU
mCCR7 shRNA	[15] Oligofactory	GGfCfCfUfCAfUfUAfUfCAfUfCfCGfUAfCfCfUfUfCAAGAGAGGfUAfCGGAfUGAfUAAfUGAGGfUfUfUfUfU
Scrambled-mCCR1 shRNA	Oligofactory	GGAfUAAGAfUGfCGfUAGAAGAfUfUfCAAGAGAfUfCfUfUfCfUAfCGfCAfUfCfUfUAfUfCfCfUfUfUfUfU
Scrambled-mCCR2-shRNA	Oligofactory	GAGfCGfUfCGfCAAfUAfCAfUfCfUGfUfUfCAAGAGAGAGAfUGfUAfUfUGfCGAfCGfCfUfUfUfUfUfUfU
Scrambled-mCCR5-shRNA	Oligofactory	GGAGfUAfUGAGAfUAfUAfCGfUfCfUGfUfUfCAAGAGAfCAGAfCGfUAfUAfUfCfUfCAfUAfCfUfUfUfUfUfU
Scrambled-mCCR7-shRNA	Oligofactory	GGAfUfCfUfCfCAfUfCGfCfCfUAfUfCfCfUAfUfUfCAAGAGAfUAGGAfUAGGfCGAfUGGAGAfUfUfUfUfUfU

Software and Algorithms		
Sigmaplot 12.5	Systat software	https://systatsoftware.com/products/sigmaplot/
FCS express 7 plus	Denovo software	https://denovosoftware.com/
Cell profiler	[16]	www.cellprofiler.com
Total Image slicer		https://www.coolutils.com/
ImageJ	[17]	https://fiji.sc/
OlyVIA Ver.2.9.1 (Build 13771)	Olympus	https://www.olympus-lifescience.com/en/support/downloads/#!dlOpen=%23detail847252030
R Package: Robust Multiarray Average procedure of the *affy* Bioconductor	[18]	
Samr R package (Significance Analysis of Microarray (SAM) algorithm)	[1]	
Molecular Signatures Database (MSigDB)		https://www.gsea-msigdb.org/gsea/msigdb
GSEA software		http://www.broadinstitute.org/gsea/index.jsp

Table S3 genes associated with PMN-MDSC, neutrophils, M1 macrophages, or M2 macrophages

Gene	Associated with	Reference
Vegfa	PMN-MDSC	19
Tnf	PMN-MDSC	21
Mpo	PMN-MDSC	22
MMP9	PMN-MDSC	21
IL6	PMN-MDSC	23
CXCL2	PMN-MDSC	21
CD274	PMN-MDSC	24
CXCL10	PMN-MDSC	21
CXCL1	PMN-MDSC	21
CD244	PMN-MDSC	21
Cebpb	PMN-MDSC	27
CCL3	PMN-MDSC	21
CCL2	PMN-MDSC	21
CCL17	PMN-MDSC	21
Arg1	PMN-MDSC	19
Ptges2	PMN-MDSC	27
Tgfb1	PMN-MDSC	27
Ddit3	PMN-MDSC	27
Csf1r	PMN-MDSC	27
CXCL14	PMN-MDSC	23
Fpr2	M1	20
Nos2	M1	25
IL12b	M1	20
CXCL9	M1	20
CD38	M1	20
CCR7	M1	20
Gpr18	M1	20
Myc	M2	20
Retnla	M2	20
Mrc1	M2	20
Egr2	M2	20
Chil3	M2	20
CD83	M2	20
Adgre1	macrophage	25
Flot2	neutrophils	26
CXCL12	neutrophils	27
Tuba4a	neutrophils	26
CXCR1	neutrophils	27
Elane	neutrophils	28
Foxo4	neutrophils	26
S100a11-ps	neutrophils	26
Lamp2	neutrophils	19
Pf4	neutrophils	27
Ifng	neutrophils	21
Icam1	neutrophils	21
CXCL13	neutrophils	23
CCL6	neutrophils	23
CXCR2	neutrophils	27
Itgb2l	neutrophils	21
Slc4a1	neutrophils	21

SUPPLEMENTAL FIGURES

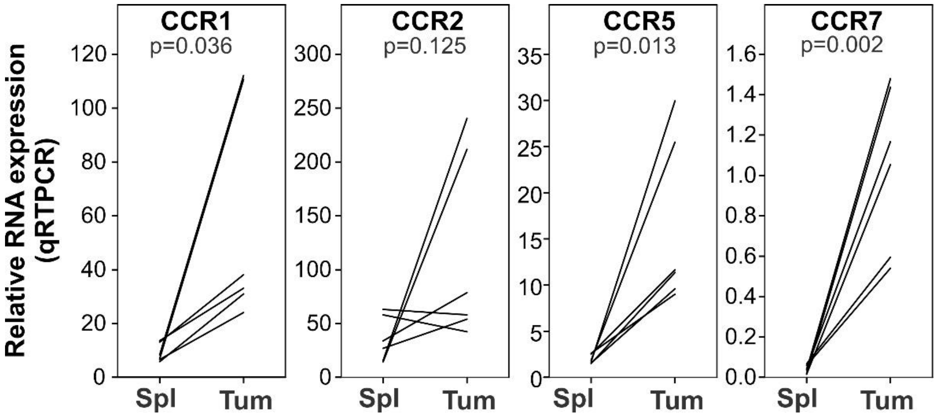

Supplemental figure 1: CCR1, CCR5, and CCR7 are upregulated in the tumor microenvironment. Expression of the indicated chemokine receptors was evaluated on $CD11b^{+}$cells magnetically isolated from the tumor or the spleen of 4T1 bearing mice.

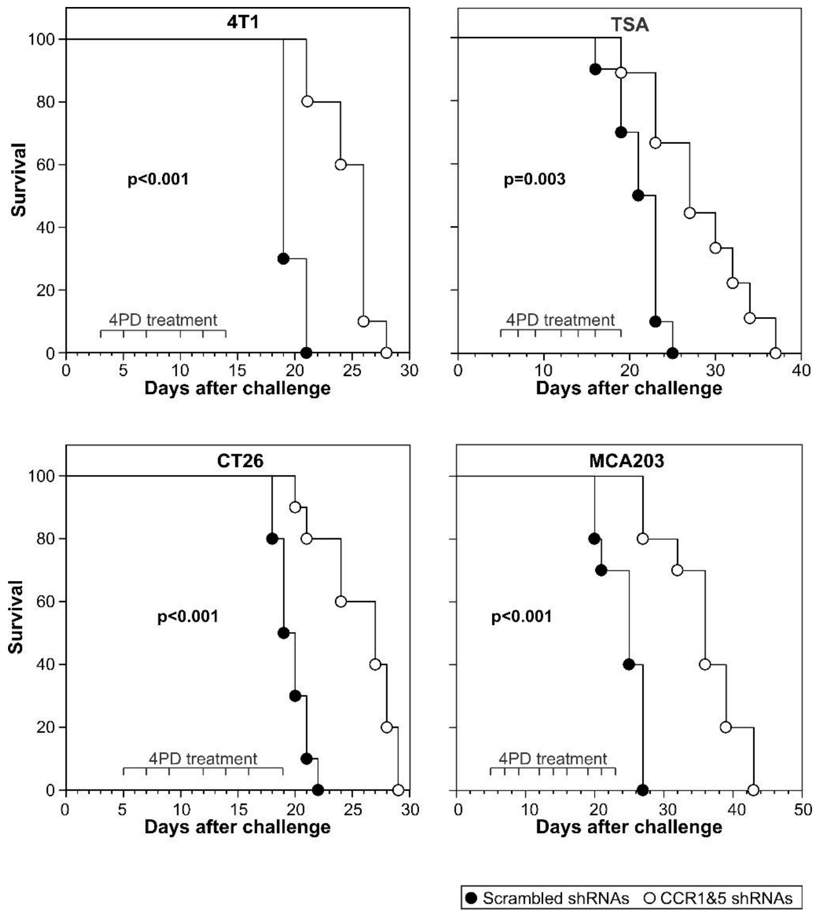

Supplemental figure 2: CCR1 and CCR5 targeted silencing delays tumor progression in multiple mouse models.

Mice (n=9-10) challenged with the indicated tumors were treated with 4PD conjugated with shRNAs specific for CCR1 and CCR5 or scrambled shRNAs once the tumors become palpable. Data derived from two independent experiments.

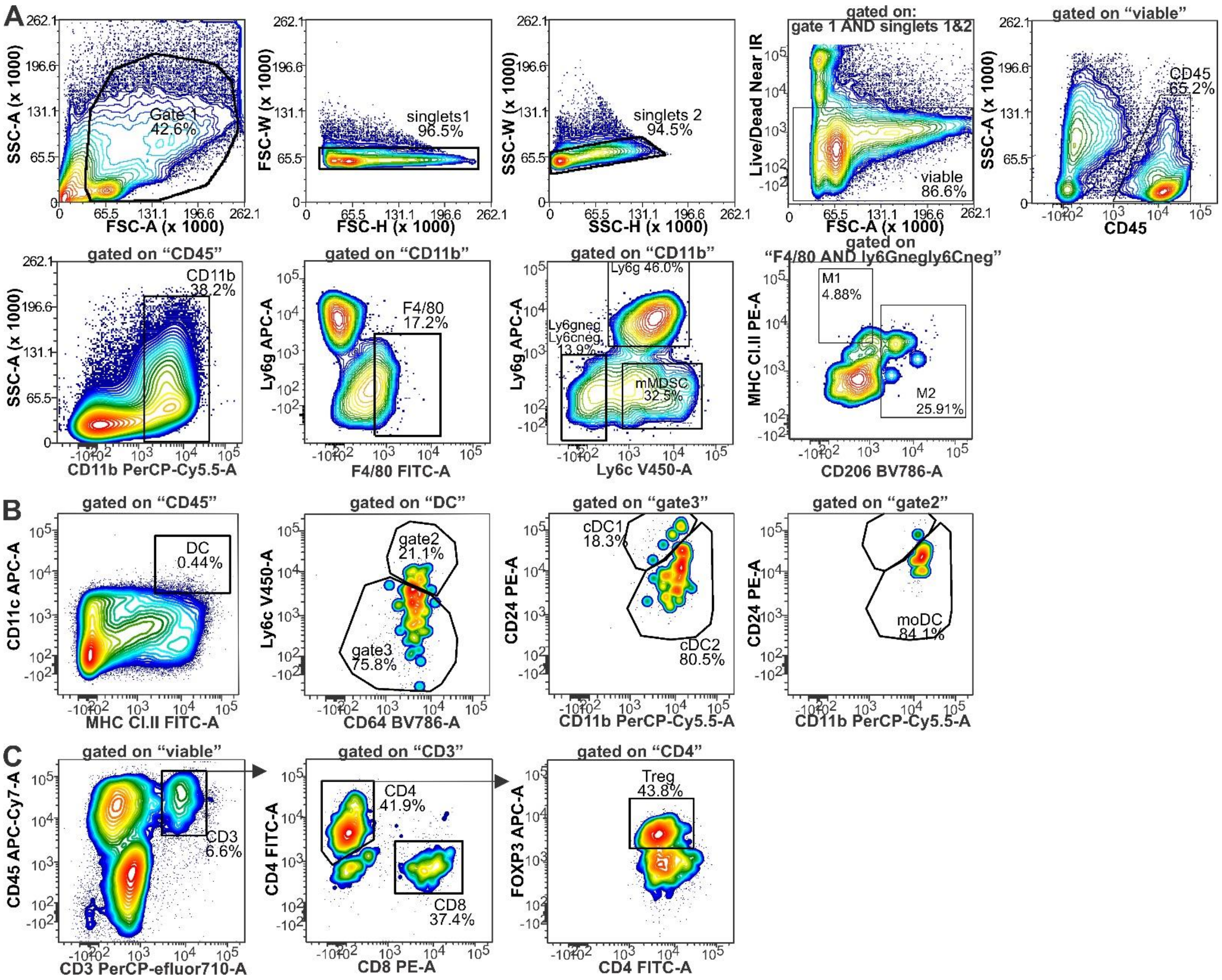

Supplemental figure 3. Gating strategy for myeloid (A), DCs (B), or T cells (C) subsets is shown and based on FMO controls.

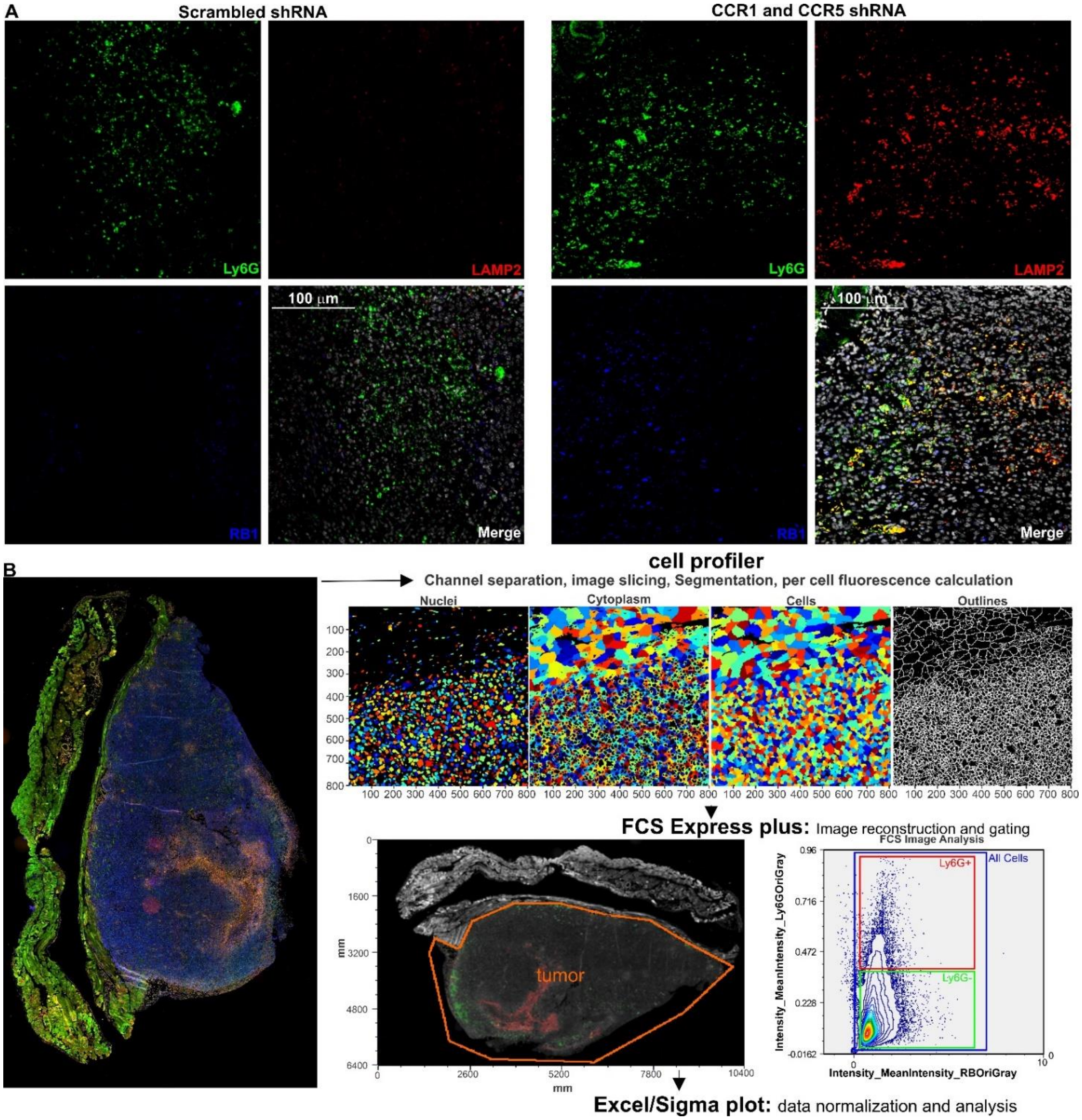

Supplemental figure 4: CCR1 and CCR5 silencing upregulated RB1 and LAMP2 in tumor infiltrating polymorphonucleate cells. 4T1 tumors from mice treated with scrambled shRNAs or CCR1 and CCR5 shRNAs were stained with DAPI and antibodies against Ly6G, RB1, and LAMP2. **B)** Scanned images from each channel of whole tumor sections were sliced in 500x500 pixel images using totalImageSlicer (https://www.coolutils.com/) and fed into cellprofiler as 8bit gray images. Primary objects (nuclei) were identified using the DAPI channel with a nuclei diameter set between 2 and 8 pixels, using the three classes Otsu Adaptive threshold method with a correction factor of 1 and the lower and upper bounds on threshold 0.1–1.0. Clumped objects were distinguished by shape and the size of the smoothing filter and minimum allowed distance between local maxima were automatically calculated. Secondary objects were identified using the autofluorescence and fluorescence of the merged image from the 3 channels acquired using the nuclei propagation method with three-classes Otzu Adaptive threshold method, 0.9 as threshold correction factor (0.0–1.0 range) and 0.02 as regularization factor. The cytoplasm as tertiary object was define as cell (secondary object) area minus the nuclei (primary object) area. For each cell, the integrated intensity mean of the DAPI channel in the nuclei and the integrated intensity mean of the Ly6G, RB1, and Ly6G channels in the cells were exported as .cpout file and analyzed using FCS Express PLUS vs7. A “tumor” gate/ROI was drawn to delineate the tumor area identified in H&E serial sections and RB1 and LAMP2 expression was evaluated within the intratumoral Ly6G^{+} and Ly6G^{-}cells. Data were normalized to the background (all cells gate) median intensity.

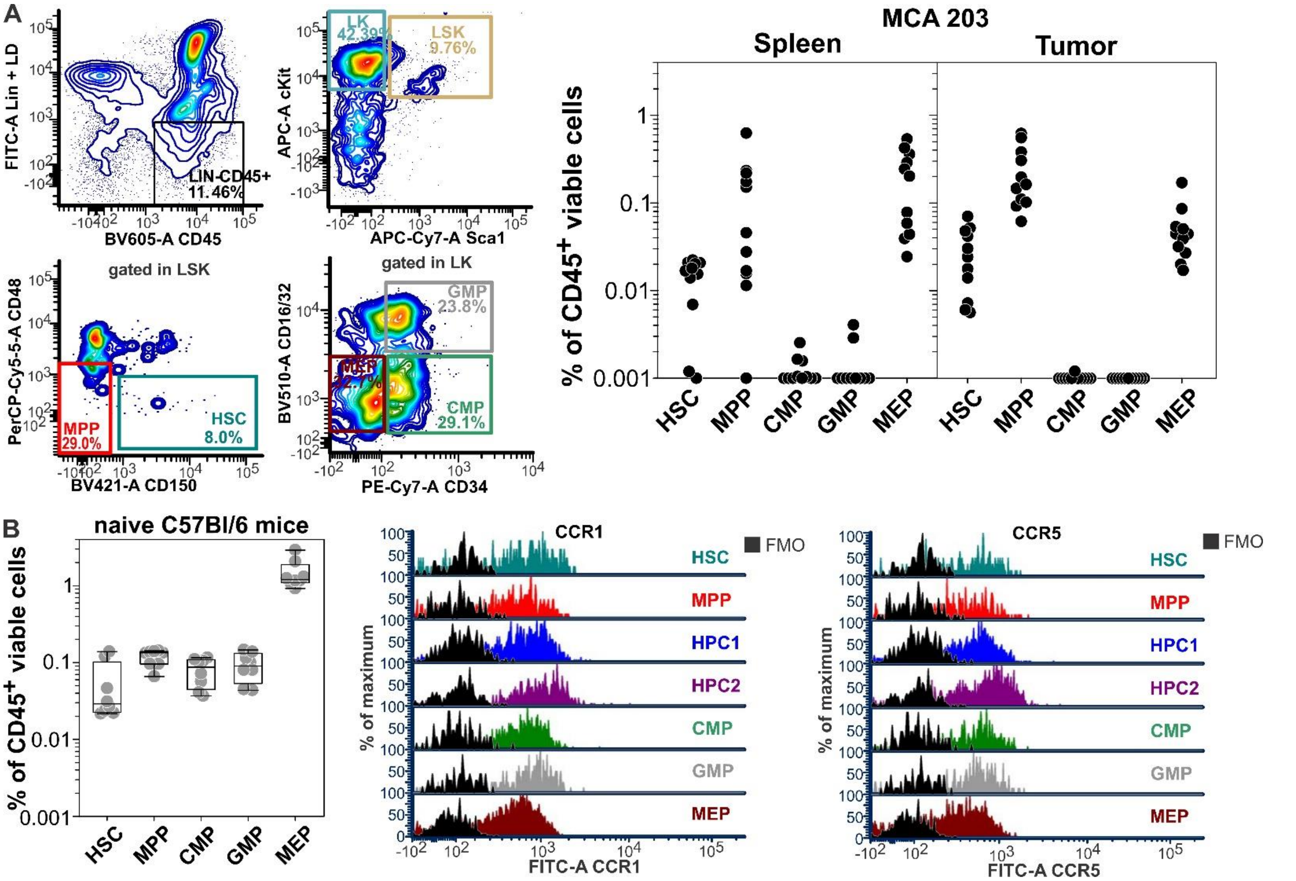

Supplemental figure 5: HSPCs express CCR1 and CCR5. A) HSPCs gating strategy and enumeration of HSPC subsets in the spleens and tumors (~0.5 cm of diameter) of mice bearing the MCA203 fibrosarcoma. **B)** Expression of CCR1 and CCR5 in the HSPC subsets from the bone marrow of naïve C57BL/6**.**

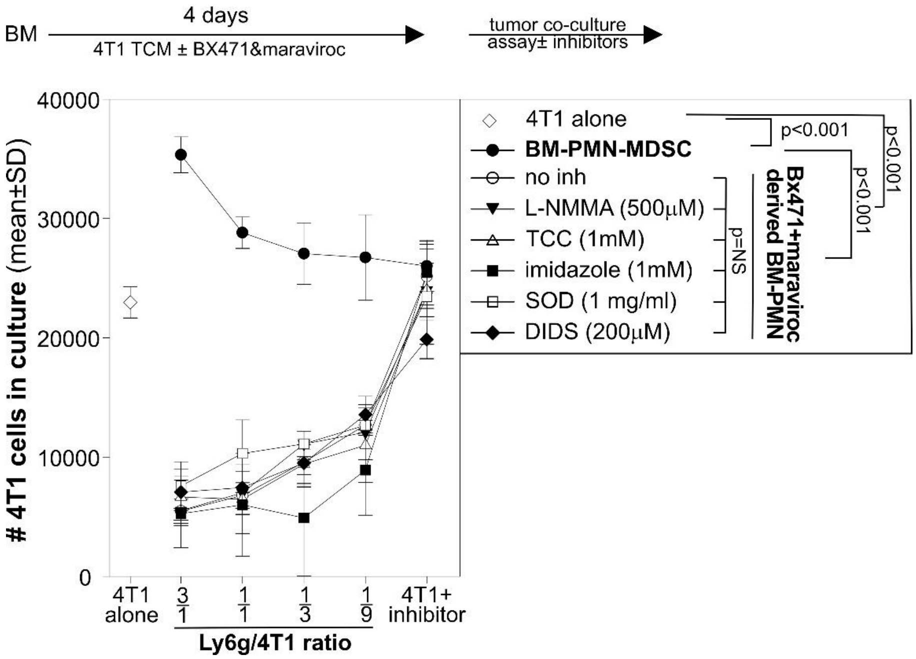

Supplemental figure 6: The main pathways involved in neutrophil cytotoxicity do not appears to mediate the observed tumoricidal activity. BM cells from naïve Balb/C mice were cultured for 4 days with 4T1-TCM in the presence or in the absence of Bx471 and Maraviroc. Flow cytometry analysis revealed that more than 90% of cells were $CD11b^{+}Ly6G^{+}$ in the “TCM + antagonists” group. $CD11b^{+}$cells were magnetically purified and cultured with 4T1-luc cells at the indicated ratio in the presence of the indicated inhibitor or vehicle. 18 hours later the number of tumor cells was determined by luciferase assay. Commercially available inhibitors were used at the reported relevant concentration. Specifically, L-NMMA was used to inhibit nitric oxide synthase 2 as in [29], N,N',N''-Triacetylchitotriose (TCC) to inhibit lysozyme as in[30], imidazole to inhibit the respiratory burst as in [31], superoxide dismutase (SOD) to inhibit superoxide action as in[32]; and 4,4′-Diisothiocyanatostil-bene-2,2′-disulfonic acid disodium salt (DIDS) to inhibit degranulation and NETosis as in[33].

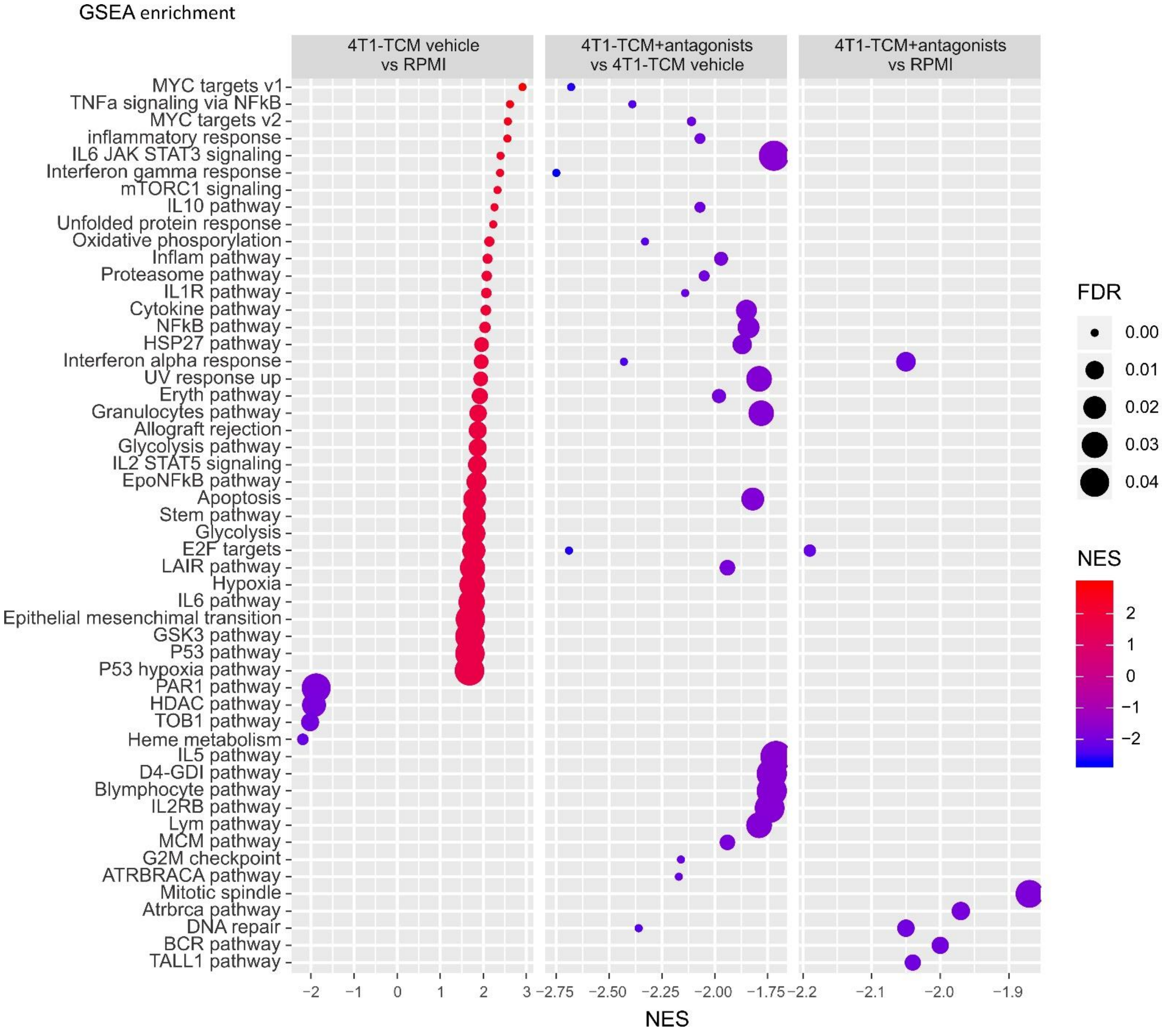

Supplementary figure 7: CCR1 and CCR5 blockade during MDSC differentiation inhibits the MDSC pathways upregulated by tumor derived factors. GSEA enrichment analysis was performed on the microarray data comparing: i) BM cells stimulated with 4T1-TCM (4T1) or complete media (RPMI), ii) BM cells stimulated with 4T1-TCM in the presence of Bx471 and Maraviroc (4T1+inhibitors) or in their absence, or iii) BM cells stimulated with 4T1-TCM in the presence of Bx471 and Maraviroc or cultured in complete media.

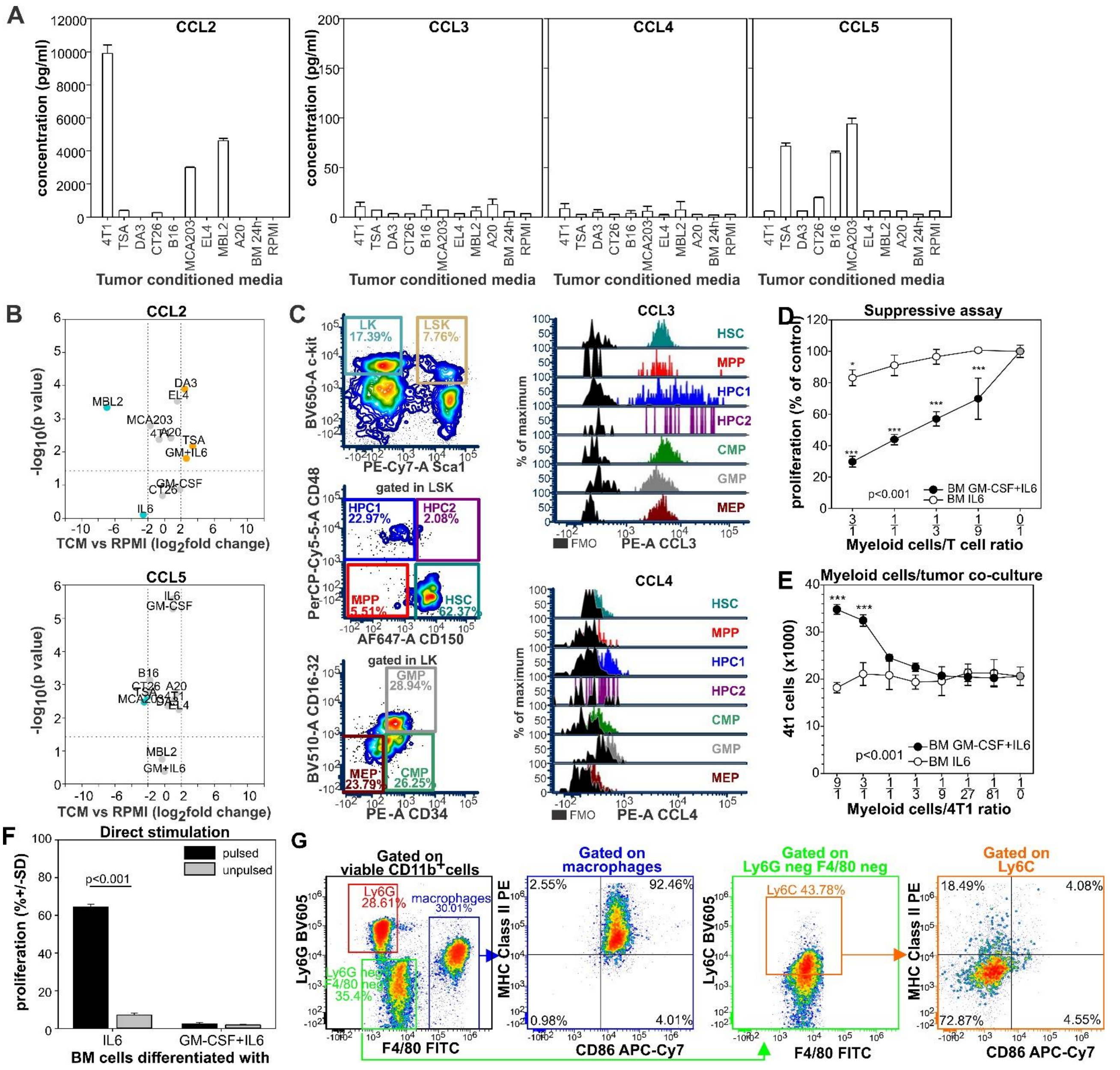

Supplemental figure 8: CCL2 and CCL5 are detectable in the TCM from different tumors but are poorly modulated in TCM stimulated BM cells whereas CCL3 and 4 are induced in different HSPC subsets.
A) CCL2, 3, 4 and 5 concentrations in the indicated tumor conditioned media were evaluated by Cytokine beads array. **B)** CCL2 and CCL5 concentrations were evaluated by cytokine beads arrays on BM cells stimulated for 24h by the indicated TCMs or indicated recombinant cytokines. The same TCMs incubated for 24h without BM cells were used as control. Supernatant from BM cells cultured in RPMI with no stimuli was used as negative control for the cultures with recombinant cytokines. Data derived from 2 independent experiments. **C)** HSPCs gating strategy and expression of CCL3 and CCL4 in BM cell subsets from C57BL/6 mice cultured for 4 hours with tumor conditioned media of the MCA203 fibrosarcoma cell line. **D, E, F)** BM cells from naïve Balb/c mice were cultured for 4 days with GM-CSF and IL6 or IL6 alone. CD11b^{+}cells were magnetically purified and **D)** tested in suppressive assays against HA specific CD8 cells stimulated with the relevant peptide; **E)** incubated with 4T1-luciferase cells for 18 hours; or **F)** pulsed with $HA_{518-526}$ peptide and incubated for 3 days with magnetically purified CD8^{+}HA-specific, CFSE-labeled T cells from Cl4 mice (pulsed myeloid cells/T cell ratio=3/1). Proliferation was evaluated by flow cytometry on viable CD3^{+}CD8^{+}cells. **G)** Flow cytometry analysis of Balb/C BM cells differentiated for 4 days by IL6 alone.

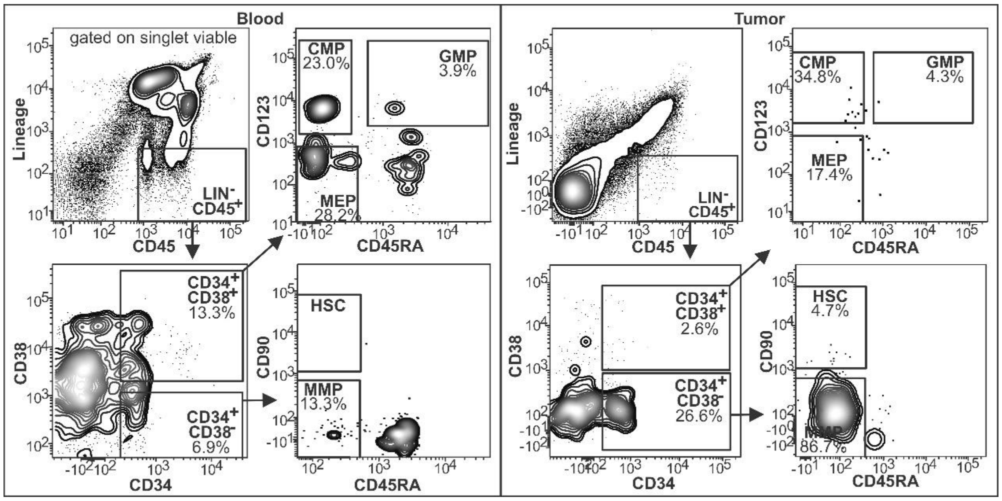

Supplemental figure 9: circulating HSPCs are present in patients with HNSCC. Gating strategy used for the enumeration of HSPCs in patients with HNSCC.

Supplemental references

1. Tusher VG, Tibshirani R, Chu G. Significance analysis of microarrays applied to the ionizing radiation response. Proc Natl Acad Sci U S A 2001;**98**(9):5116-21 doi: 10.1073/pnas.091062498.
2. Serafini P, Meckel K, Kelso M, et al. Phosphodiesterase-5 inhibition augments endogenous antitumor immunity by reducing myeloid-derived suppressor cell function. J Exp Med 2006;**203**(12):2691-702 doi: 10.1084/jem.20061104.
3. Pulaski BA, Ostrand-Rosenberg S. Mouse 4T1 breast tumor model. Curr Protoc Immunol 2001;**Chapter 20**:Unit 20 2 doi: 10.1002/0471142735.im2002s39.
4. Brattain MG, Strobel-Stevens J, Fine D, et al. Establishment of mouse colonic carcinoma cell lines with different metastatic properties. Cancer Res 1980;**40**(7):2142-6
5. Nanni P, de Giovanni C, Lollini PL, et al. TS/A: a new metastasizing cell line from a BALB/c spontaneous mammary adenocarcinoma. Clin Exp Metastasis 1983;**1**(4):373-80 doi: 10.1007/BF00121199.
6. Barth RJ, Jr., Bock SN, Mule JJ, et al. Unique murine tumor-associated antigens identified by tumor infiltrating lymphocytes. J Immunol 1990;**144**(4):1531-7
7. Bronte V, Cingarlini S, Apolloni E, et al. Effective genetic vaccination with a widely shared endogenous retroviral tumor antigen requires CD40 stimulation during tumor rejection phase. J Immunol 2003;**171**(12):6396-405 doi: 10.4049/jimmunol.171.12.6396.
8. Matory YL, Chen M, Dorfman DM, et al. Antitumor activity of three mouse mammary cancer cell lines after interferon-gamma gene transfection. Surgery 1995;**118**(2):251-5; discussion 55-6 doi: 10.1016/s0039-6060(05)80331-9.
9. Thomas GR, Chen Z, Oechsli MN, et al. Decreased expression of CD80 is a marker for increased tumorigenicity in a new murine model of oral squamous-cell carcinoma. International Journal of Cancer 1999;**82**(3):377-84 doi: 10.1002/(sici)1097-0215(19990730)82:3<377::Aid-ijc11>3.0.Co;2-9.
10. Kang Y, Siegel PM, Shu W, et al. A multigenic program mediating breast cancer metastasis to bone. Cancer Cell 2003;**3**(6):537-49 doi: 10.1016/s1535-6108(03)00132-6.
11. Gao JL, Wynn TA, Chang Y, et al. Impaired host defense, hematopoiesis, granulomatous inflammation and type 1-type 2 cytokine balance in mice lacking CC chemokine receptor 1. J Exp Med 1997;**185**(11):1959-68 doi: 10.1084/jem.185.11.1959.
12. Ishida N, Hayashi K, Hattori A, et al. CCR1 acts downstream of NFAT2 in osteoclastogenesis and enhances cell migration. J Bone Miner Res 2006;**21**(1):48-57 doi: 10.1359/JBMR.051001.
13. Leuschner F, Dutta P, Gorbatov R, et al. Therapeutic siRNA silencing in inflammatory monocytes in mice. Nat Biotechnol 2011;**29**(11):1005-10 doi: 10.1038/nbt.1989.
14. Kim SS, Peer D, Kumar P, et al. RNAi-mediated CCR5 silencing by LFA-1-targeted nanoparticles prevents HIV infection in BLT mice. Mol Ther 2010;**18**(2):370-6 doi: 10.1038/mt.2009.271.
15. Shields JD, Kourtis IC, Tomei AA, et al. Induction of lymphoidlike stroma and immune escape by tumors that express the chemokine CCL21. Science 2010;**328**(5979):749-52 doi: 10.1126/science.1185837.
16. Carpenter AE, Jones TR, Lamprecht MR, et al. CellProfiler: image analysis software for identifying and quantifying cell phenotypes. Genome Biol 2006;**7**(10):R100 doi: 10.1186/gb-2006-7-10-r100.
17. Schindelin J, Arganda-Carreras I, Frise E, et al. Fiji: an open-source platform for biological-image analysis. Nat Methods 2012;**9**(7):676-82 doi: 10.1038/nmeth.2019.
18. Bolstad BM, Irizarry RA, Astrand M, et al. A comparison of normalization methods for high density oligonucleotide array data based on variance and bias. Bioinformatics 2003;**19**(2):185-93 doi: 10.1093/bioinformatics/19.2.185.
19. Zilio S, Serafini P. Neutrophils and Granulocytic MDSC: The Janus God of Cancer Immunotherapy. Vaccines (Basel) 2016;**4**(3):31 doi: 10.3390/vaccines4030031.
20. Jablonski KA, Amici SA, Webb LM, et al. Novel Markers to Delineate Murine M1 and M2 Macrophages. PLoS One 2015;**10**(12):e0145342 doi: 10.1371/journal.pone.0145342.
21. Fridlender ZG, Sun J, Mishalian I, et al. Transcriptomic analysis comparing tumor-associated neutrophils with granulocytic myeloid-derived suppressor cells and normal neutrophils. PLoS One 2012;**7**(2):e31524 doi: 10.1371/journal.pone.0031524.
22. Youn JI, Collazo M, Shalova IN, et al. Characterization of the nature of granulocytic myeloid-derived suppressor cells in tumor-bearing mice. J Leukoc Biol 2012;**91**(1):167-81 doi: 10.1189/jlb.0311177.
23. Shaul ME, Levy L, Sun J, et al. Tumor-associated neutrophils display a distinct N1 profile following TGFbeta modulation: A transcriptomics analysis of pro- vs. antitumor TANs. Oncoimmunology 2016;**5**(11):e1232221 doi: 10.1080/2162402X.2016.1232221.

24. Lu C, Redd PS, Lee JR, et al. The expression profiles and regulation of PD-L1 in tumor-induced myeloid-derived suppressor cells. Oncoimmunology 2016;**5**(12):e1247135 doi: 10.1080/2162402X.2016.1247135.
25. Sica A, Bronte V. Altered macrophage differentiation and immune dysfunction in tumor development. J Clin Invest 2007;**117**(5):1155-66 doi: 10.1172/JCI31422.
26. Ko J, Rayman PA, Yang Y, et al. Differential gene expression in G-MDSC and neutrophils from renal cell carcinoma patients. Journal for ImmunoTherapy of Cancer 2014;**2**(S3):P216-P16 doi: 10.1186/2051-1426-2-s3-p216.
27. Zhou J, Nefedova Y, Lei A, et al. Neutrophils and PMN-MDSC: Their biological role and interaction with stromal cells. Semin Immunol 2018;**35**:19-28 doi: 10.1016/j.smim.2017.12.004.
28. Elpek KG, Cremasco V, Shen H, et al. The tumor microenvironment shapes lineage, transcriptional, and functional diversity of infiltrating myeloid cells. Cancer Immunol Res 2014;**2**(7):655-67 doi: 10.1158/2326-6066.CIR-13-0209.
29. Bronte V, Serafini P, De Santo C, et al. IL-4-induced arginase 1 suppresses alloreactive T cells in tumor-bearing mice. J Immunol 2003;**170**(1):270-8 doi: 10.4049/jimmunol.170.1.270.
30. Attri P, Kaushik NK, Kaushik N, et al. Plasma treatment causes structural modifications in lysozyme, and increases cytotoxicity towards cancer cells. Int J Biol Macromol 2021;**182**:1724-36 doi: 10.1016/j.ijbiomac.2021.05.146.
31. Kantar A, Oggiano N, Gabbianelli R, et al. Effect of Imidazole Salicylate on the Respiratory Burst of Polymorphonuclear Leukocytes. Current Therapeutic Research-Clinical and Experimental 1993;**54**(2):241-47 doi: Doi 10.1016/S0011-393x(05)80607-1.
32. Bowler RP, Nicks M, Tran K, et al. Extracellular superoxide dismutase attenuates lipopolysaccharide-induced neutrophilic inflammation. Am J Respir Cell Mol Biol 2004;**31**(4):432-9 doi: 10.1165/rcmb.2004-0057OC.
33. Maueroder C, Mahajan A, Paulus S, et al. Menage-a-Trois: The Ratio of Bicarbonate to CO2 and the pH Regulate the Capacity of Neutrophils to Form NETs. Front Immunol 2016;**7**(583):583 doi: 10.3389/fimmu.2016.00583.
