# Supplementary material for: CCR1 and CCR5 mediate cancer-induced myelopoiesis and differentiation of myeloid cells in the tumor
Source: J Immunother Cancer. 2022 Jan 21;10(1):e003131. doi: 10.1136/jitc-2021-003131 (PMC8785210; doi:10.1136/jitc-2021-003131)
Supplement: Supplementary data [file jitc-2021-003131supp003.pdf]

CCR1 and CCR5 mediate cancer driven myelopoiesis and MDSC differentiation.

Serena Zilio, Silvio Bicciato, Donald Weed, and Paolo Serafini·

Graphical Abstract

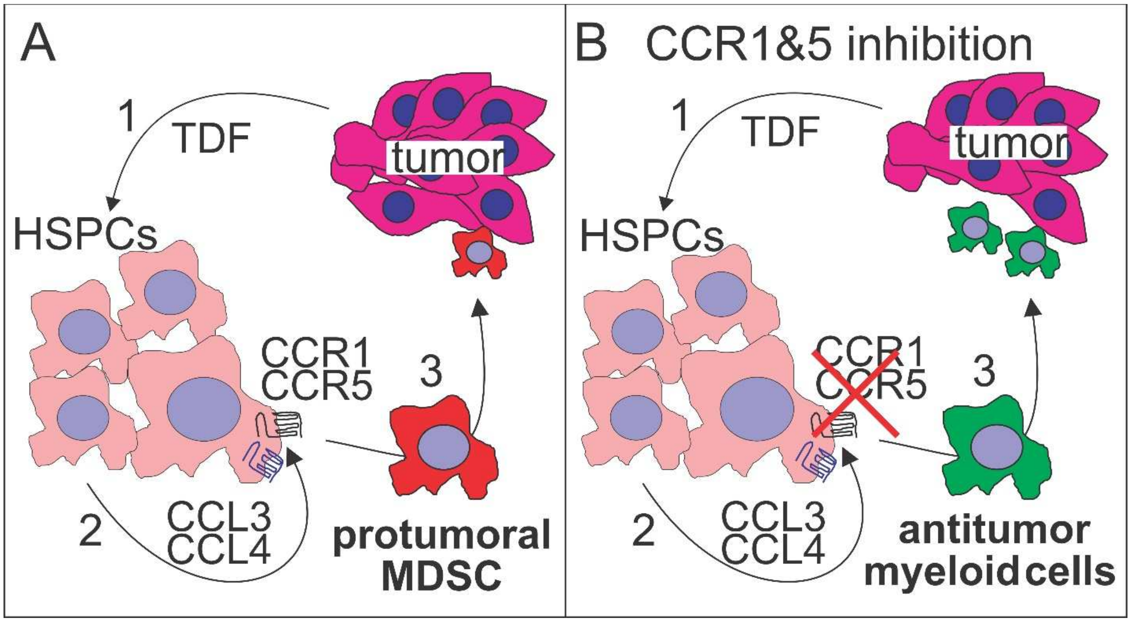

A) Tumor derived factors (1) induce HSPCs to upregulate and secrete of CCL3 and CCL4 (2). These chemokines autocrinally bind to CCR1 and CCR5 and activate their differentiation into MDSCs (3) favoring tumor progression and metastasis. **B)** Inhibition of CCR1 and CCR5 signaling blocks HSPCs conversion in MDSCs, restores the default differentiation of myeloid cells with anti-tumor activity, and reduces tumor progression.
